# Thermodynamics Evaluation of Selective Hydride Reduction for α,β-Unsaturated Carbonyl Compounds

**DOI:** 10.3390/molecules28062862

**Published:** 2023-03-22

**Authors:** Bao-Long Chen, Sha Jing, Xiao-Qing Zhu

**Affiliations:** The State Key Laboratory of Elemento-Organic Chemistry, Collaborative Innovation Center of Chemical Science and Engineering, College of Chemistry, Nankai University, Tianjin 300071, China; jing_sha@wuxiapptec.com

**Keywords:** α, β-unsaturated carbonyl compounds, hydride affinity, selective reduction, reduction potential

## Abstract

The selective reduction of α,β-unsaturated carbonyl compounds is one of the core reactions and also a difficult task for organic synthesis. We have been attempting to study the thermodynamic data of these compounds to create a theoretical basis for organic synthesis and computational chemistry. By electrochemical measurement method and titration calorimetry, in acetonitrile at 298 K, the hydride affinity of two types of unsaturated bonds in α,β-unsaturated carbonyl compounds, their single-electron reduction potential, and the single-electron reduction potential of the corresponding radical intermediate are determined. Their hydrogen atom affinity, along with the hydrogen atom affinity and proton affinity of the corresponding radical anion, is also derived separately based on thermodynamic cycles. The above data are used to establish the corresponding “Molecule ID Card” (Molecule identity card) and analyze the reduction mechanism of unsaturated carbonyl compounds. Primarily, the mixture of any carbonyl hydride ions and Ac-tempo^+^ will stimulate hydride transfer process and create corresponding α,β-unsaturated carbonyl compounds and Ac-tempoH from a thermodynamic point of view.

## 1. Introduction

The famous Michael addition reaction [1,2,3,4,5], Robinson annulation reaction [6,7,8,9,10], and Diels–Alder reaction [11,12,13,14,15] in organic synthesis include the participation of α,β-unsaturated carbonyl compounds, which is one of the most effective methods to construct carbon chains and ring structures. In addition, the reduction of α,β-unsaturated carbonyl compounds is generally used to prepare saturated aldehyde ketones, allyl alcohol, saturated alcohol, and other important compounds, and the control over the reduction sites of such compounds has become a research hotspot for organic chemists [16,17,18,19,20,21].

**Scheme 1:**
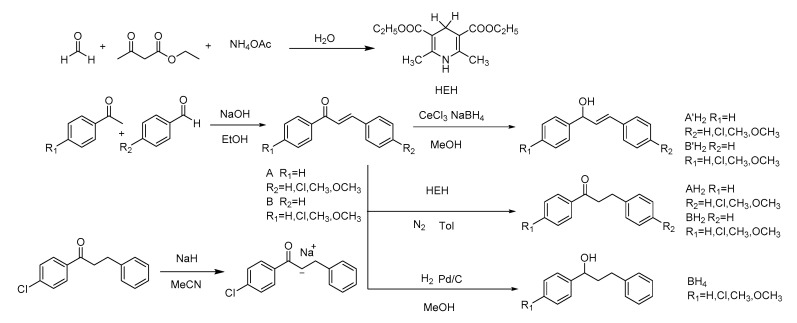
The compounds synthesized in this work.

The polarity of the carbonyl group of α,β-unsaturated carbonyl compounds and the alternate distribution of conjugated chain charges give carbonyl carbon and β-carbon certain positive charges, while carbonyl oxygen and α-carbon have certain negative charges. Such phenomena lead to amphipathic characteristics: nucleophile can be added to carbonyl carbon to produce 1, 2 addition reaction (Grignard reagent as “hard” nucleophile), or added to β-carbon to produce 1, 4 addition reaction (lithium dimethylcuprate as “soft” nucleophile). Therefore, chemical selectivity of reduction reactions is not only the focus of theoretical research but also the difficulty of industrial application. Previous studies [16,17,18,19,20,21] can only control the type of reaction by screening the reducing agent, optimizing the reaction conditions and adding catalysts, but this paper studies the selective reduction of α,β-unsaturated carbonyl compounds from the perspective of thermodynamics. The main purpose of this work is to quantify the hydrogenation process by establishing a database and theoretical foundation. In this paper, the following seven series of compounds in Scheme 1 have been designed and synthesized. All compounds have been reported, and we will not present them as new. It is noteworthy that A’H_2_ and B´H_2_ are synthesized for the first time by the CeCl_3_ method mentioned below. By measuring their five thermodynamic parameters, we have analyzed the energy changes of elementary reactions at each step and finally determined the specific pathways of hydrogen transfer, thereby providing theoretical support for the selective reduction of α,β-unsaturated carbonyl compounds.

We have determined the hydride affinity (Equations (1)–(3)) of the seven series of compounds (Scheme 1). In Equation (3), X represents the structure of a compound that can accept a hydride anion. Since there are two types of unsaturated bonds in α,β-unsaturated carbonyl compounds, and both the double bond and carbonyl group can accept hydride anions, the hydride affinity of carbonyl groups accepting hydride anions in 2-positions is denoted herein as ∆*H*_H_-_A_^2^, while the hydride affinity of the double bond accepting hydride anions in 4-positions is denoted herein as ∆*H*_H_-_A_^4^.

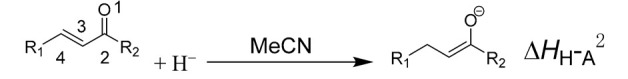
(1)

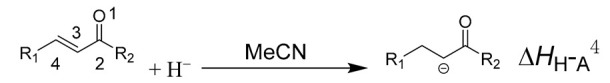
(2)

∆*H*_H_-_A_(X) = *H*_f_ (XH) − [*H*_f_ (X) + *H*_f_ (H^−^)]. (3)

**Scheme 2:**
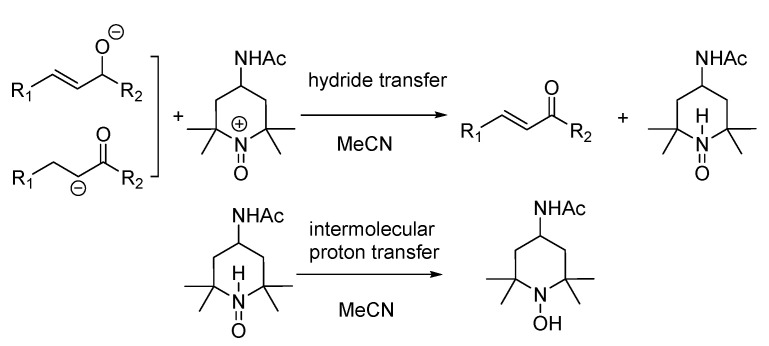
The hydride transfer reactions in this work.

Since the free hydride ion in acetonitrile is not available, the process of unsaturated compounds accepting hydride anion is exactly opposite to the heterolytic process of anions (Equation (4)). The thermodynamic driving force of hydride transfer depends on not only the ability of the hydride donor to provide the hydride but also the ability of the hydride acceptor to accept the hydride. However, the hydride affinity of X in solution, ∆*H*_H_-_A_(X), can be obtained from the reaction enthalpy change of the corresponding carbanions XH with a strong hydride acceptor, such as 4- acetylamino-2,2,6,6-tetramethylpiperidine1-oxoammonium (Ac-tempo^+^) [22,23] (Equation (5)). The enthalpy change of hydride transfer and proton transfer in the molecular (Ac-tempo^+^ ClO_4_^–^) were combined: ∆*H* (Ac-tempo^+^) = ∆*H*_H_-_A_(Ac-tempo^+^) + ∆*H*_IPT_(Ac-tempo^+^) (Scheme 2).

∆*H*_H_-_A_(X) = −∆*H*_H_-_D_(XH) (4)

∆*H*_H_-_A_(X) = ∆*H* (Ac-tempo^+^) − ∆*H*_rxn_ (5)

A hydride anion consists of a proton and two electrons, so there are three pathways available for hydride transfer: (1) one-step hydride anion transfer; (2) hydrogen atom-electron transfer; (3) electron-initiated e-H^+^-e or e-H^•^ multi-step transfer. The same is true for the acceptance of hydride by unsaturated carbonyl compounds. General hydride anion transfer mainly involves the following bond energy changes.

In this work, in acetonitrile at 298 K, hydride affinity, ∆*H*_H_-_A_(X), is defined as the molar enthalpy change of X capturing a hydride anion. Hydrogen atom affinity, ∆*H*_HA_(X), is defined as the molar enthalpy change of X capturing a hydrogen atom. Hydrogen atom affinity, ∆*H*_HA_(X^•−^), is defined as the molar enthalpy change of a radical anion X^•−^ capturing a hydrogen atom. Proton affinity, ∆*H*_PA_ (X^•−^), is defined as the molar enthalpy change of a radical anion X^•−^ capturing a proton.

**Figure 1:**
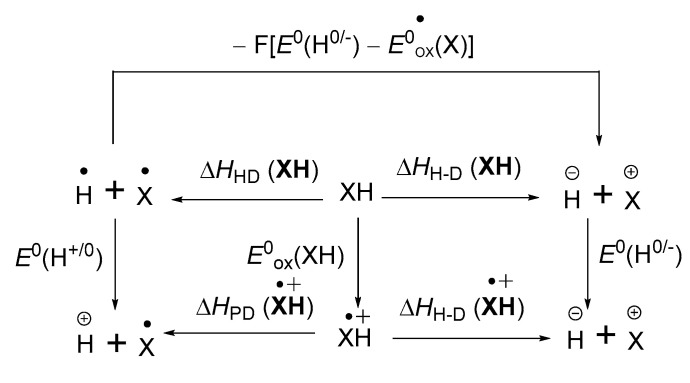
Thermodynamic cycles between compounds and hydrogen.

The thermodynamic parameters of reactants and reaction intermediates (Figure 1) may be detected based on the electrochemical and calorimetric data, and the corresponding derivation equations are shown in Equations (6)–(8). *E*^0^_red_(X) is the reduction potential of compound A, AH2, and A’H2. *E*^0^red(XH^•^) is the reduction potential of intermediates of compound A, AH_2_, and A’H_2_ gaining hydrogen atoms (Figure 1). We have replaced ∆*H* with ∆*G*_ET_ and referred to the literature value [24] for determining reversible potentials taking *E*_1/2_(H^+/0^) = −2.307 (V vs Fc^+/0^), *E*_1/2_(H^0/−^) = −1.137 (V vs Fc^+/0^) (Fc = ferrocene). F stands for Faraday’s constant (23.05 kcal mol^−1^ V^−1^).

∆*H*_HA_ (X) = ∆*H*_H_-_A_ (X) + F [*E*^0^(H^0/−^) − *E*^0^_red_(XH**^•^**)] (6)

∆*H*_HA_ (X^•−^) = ∆*H*_H_-_A_ (X) - F [*E*^0^(H^0/−^) − *E*^0^_red_(X)] (7)

∆*H*_PA_ (X^•−^) = ∆*H*_HA_ (X^•−^) + F [*E*^0^(H^0/+^) − *E*^0^_red_(XH**^•^**)] (8)

## 2. Results

**Figure 2:**
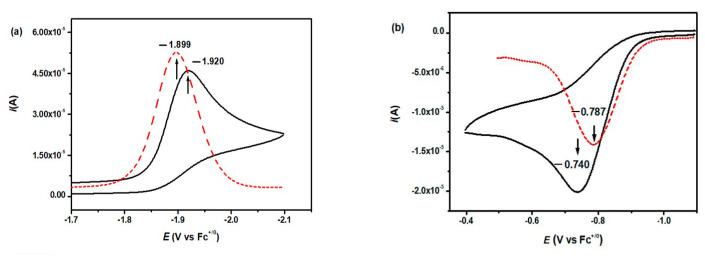
Cyclic voltammetry (CV) and Osteryoung square wave voltammetry (OSWV) of unsaturated ketone A(R = OCH_3_) (**a**) and an anion accepting a hydride anion at position 4 A(R = OCH_3_) (**b**) in deaerated acetonitrile containing 0.1 M n-Bu_4_NPF_6_ as supporting electrolyte. Solid line: CV graph (sweep rate = 0.1 V/s); dashed line: OSWV graph. The CV values are cathodic peak potentials if the CV figure does not have peaks and troughs. CV and OSWV graphs all have two troughs s or peaks, and one part s is visible.

Common electrochemical measurement method mainly includes cyclic voltammetry (CV) and Osteryoung square wave voltammetry (OSWV). In this study, we have used these methods to determine the single-electron reduction potentials of three types of unsaturated carbonyl compounds (Scheme 1) and the reduction potentials of radical intermediates corresponding to unsaturated carbonyl compounds in Figure 2 and Supporting Information. The obtained data is listed in Table 1. These data were calculated based on data obtained via the OSWV method.

**Figure 3:**
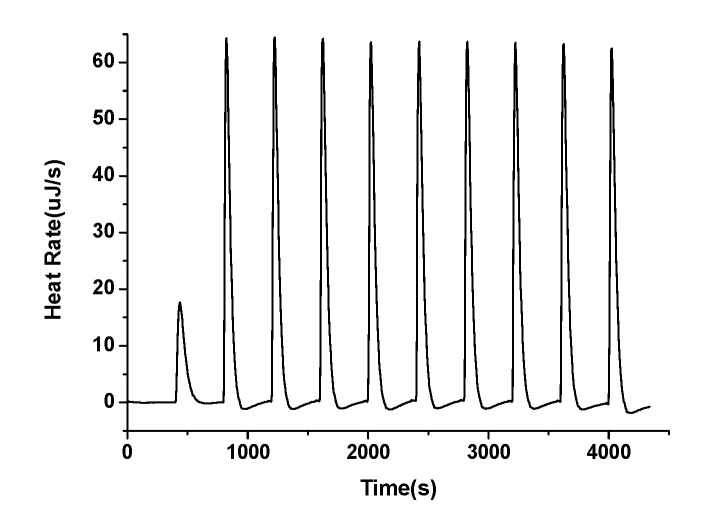
ITC (Isothermal titration calorimetry) for determining the heat of the reaction between Ac-tempo^+^ClO_4_^−^ and BH_4_ (R = CH_3_) in acetonitrile at 298 K. Titration was conducted by adding 10 μL of Ac-tempo^+^ClO_4_^−^(1.1 mM) every 400 s into the acetonitrile containing the BH_4_(R=CH_3_) (10 mM).

∆*H*rxn is the molar enthalpy change of the reaction (Equation (5)) in acetonitrile, which can be determined by using titration calorimetry. In our previous work, we first determined the molar enthalpy change (∆*H*rxn) of the reaction between Ac-tempo^+^ClO_4_^–^ and BNAH directly by isothermal titration calorimetry (ITC), calibrated the hydride affinity of Ac-tempo^+^ClO_4_^–^ −∆*H* (Ac-tempo^+^) to −105.60 kcal/mol. We determined the molar enthalpy change (∆*H*rxn) of the reaction between the anions (BH_4_) and Ac-tempo^+^ClO_4_^–^ (Figure 3 and Supporting Information), relevant data are listed in Table 1.

Based on the electrochemical data *E*_1/2_(H^+/0^) = −2.307 (V vs. Fc^+/0^) and *E*_1/2_(H^0/−^) = −1.137 (V vs. Fc^+/0^) and ∆*H*(Ac-tempo^+^), we substituted the thermodynamic data (directly measured) into Equations (4)–(7) to obtain the hydride affinity ∆*H*_H_-_A_(X) and hydrogen-atom affinity ∆*H*_HA_(X) of unsaturated carbonyl compounds as well as the hydrogen–atom affinity ∆*H*_HA_(X^•−^) and proton affinity ∆*H*_PA_(X^•−^) of the corresponding radical anions. All the data are summarized in Table 2.

## 3. Discussion

### 3.1. Hydride Affinity of α,β-Unsaturated Carbonyl Compounds

#### 3.1.1. Hydride Affinity of Carbon–Oxygen Double Bond in α,β-Unsaturated Carbonyl Compounds

Table 2 indicates that the hydride affinity of the carbonyl group in these three types of compounds is generally small, with absolute values roughly distributing in the range of −18.77–−52.74 kcal/mol. The corresponding oxygen anions formed by carbonyl compounds accepting hydride anions are good hydride donors, which can reduce most hydride acceptors. However, hydride donors with moderate strength such as BNAH (64.2 kcal/mol) or HEH (69.3 kcal/mol) [25] can barely reduce the carbonyl groups in α,β-unsaturated ketones or saturated ketones.

In comparison of the hydride affinity of carbonyl groups in three types of unsaturated compounds with the same distal substituent (R ≠ H), these data are found to increase in this order: |∆*H*_H_-_A_(A)| > |∆*H*_H_-_A_(B)| > |∆*H*_H_-_A_(AH_2_)|. The absolute value of the hydride affinity of the saturated carbonyl group is much smaller than that of two unsaturated carbonyl compounds (A and B); therefore, the presence of a conjugated structure increases the molecular stability, and alcohol anion attached double bone is more unstable than oxygen anions and more likely to lose a hydride anion.

A comparison of B and BH_2_ with the same substituent shows that the value of |∆*H*_H_-_A_(A)-∆*H*_H_-_A_(AH_2_)| (conjugation energy) is from 11.63 to 15.54 kcal/mol, and electron-donating groups increase this difference. Since benzyl can still transmit the substituent effect of the benzene ring para-position, the hydride affinity varies as much for BH_2_ as for A and B when the substituent is not conjugated with the carbonyl. 

The carbonyl groups in A and B are all in the conjugated system, and the change of the substituent position may cause a slight effect on the hydride affinity of the carbonyl group in the unsaturated system. Therefore, the difference between the two values is small (approximately 2 kcal/mol).

#### 3.1.2. Hydride Affinity of Carbon–Carbon Double Bond in α,β-Unsaturated Carbonyl Compounds

Table 2 indicates that ∆*H*_H_-_A_(X) of the C=C double bond in the conjugated system is in the range of −27.66~−40.08 kcal/mol. The absolute value of ∆*H*_H_-_A_(A)_4_ (the hydride affinity for the carbon–carbon double bond of A) is higher than that of ∆*H*_H_-_A_(B)_4_ of B, and the absolute value of their difference is slightly higher than the absolute value of the difference in hydride affinity between corresponding carbonyl compounds (Table 2). This is due to the fact that the distance between the substituent and the reaction site in A is shorter than that in B, and the distance between the substituent and the active center affects the corresponding reactivity; the closer the distance is, the greater the effect will be.

A comparison of hydride affinity between carbon–carbon double bond in α,β-unsaturated carbonyl compounds and benzylic carbocation (e.g., hydride affinities of 4-CH_3_OC_6_H_4_CH_2_^+^, 4-MeC_6_H_4_CH_2_^+^, C_6_H_4_CH_2_^+^, and 4-ClC_6_H_4_CH_2_^+^ are −107, −112, −118, and −123 kcal/mol, respectively [26]), shows that the absolute value of the former is much smaller than that of latter. Because the latter releases energy by forming a new C-H σ- bond, while the former involves not only the formation process of the C-H σ- bond, but also the breaking process of the C=C π- bond, which requires a large amount of absorbed energy.

#### 3.1.3. Selective Reduction of α,β-Unsaturated Carbonyl Compound

To quantitatively examine the selectivity of the hydride addition process, two types of hydride affinities analyzed, and the following inferences are drawn.

**Figure 4:**
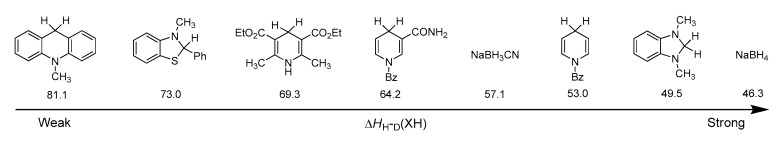
Hydricity of some typical hydride donors to release a hydride anion in acetonitrile.

**Scheme 3:**
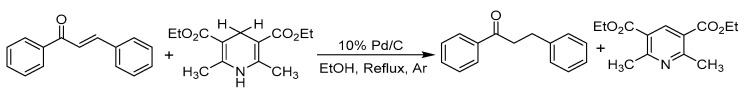
Synthesis of saturated ketones.

First, the conjugated system makes these compounds (A and B) relatively stable, while the unsaturated bonds are relatively weak in accepting hydride ions, and they work in a range of −27.66~−52.74 kcal/mol. It is difficult for BNAH, HEH, etc., to reduce carbonyl groups or carbon–carbon double bonds in α,β-unsaturated carbonyl compounds. The energy released by any hydride acceptor to bond with a hydride ion is not sufficient to compensate for the energy consumed by any hydride donor to dissociate a hydride ion. Therefore, the reaction may be induced by using strong reducing agents (Figure 4) including NaBH_4_, LiAlH_4_ [27], etc., or by changing the reaction conditions after the addition of catalysts [28]. In this study, HEH is used as the hydride donor to reduce α,β-unsaturated carbonyl compounds (Scheme 3), Pd/C catalyst is added, and the reaction is carried out under the heating reflux condition.

Second, the absolute value of the hydride affinity of the carbonyl group is generally larger than that of the corresponding carbon–carbon double bond. Thermodynamically, the carbonyl group is better than the double bond, that is, the carbonyl group is reduced first. However, their difference is not large, so the selective reduction of such a molecule is only marginally controllable.

Third, oxygen contains a lone pair of electrons and is more electronegative than carbon in an unsaturated system. Adding protic or Lewis acids and metal ions to change the polarity of the carbonyl group may induce the reaction to occur in a directional manner. For example, the hydride affinity of NaBH_4_ is close to that of both types of unsaturated bonds in the unsaturated system. However, the electron deficiency of B makes this hydride donor easily bond with electronegative O atoms [29], so NaBH_4_ mainly demonstrates 1,2- position addition during the reduction of α,β-unsaturated carbonyl compounds, or the reaction site may be activated by adding CeCl_3_ [30].

Finally, the selectivity of the reduction reaction can be improved by introducing electron-withdrawing substituents. As the electron-withdrawing capability of the substituent increases, the difference between the hydride affinities of the carbonyl group and the C=C bond of α,β-unsaturated carbonyl compounds increases.

### 3.2. Hydrogen–Atom Affinity of α,β-Unsaturated Carbonyl Compounds

Studies on the hydrogen–atom affinity lead to the following inferences.

First, the hydrogen atom affinity ∆*H*_HA_(X) of the three types of carbonyl compounds is in the range of −4.93~−40.61 kcal/mol, indicating that they are all very weak hydrogen atom acceptors.

Second, in the three types of compounds, |∆*H*_H_-_A_(X)| is higher than the corresponding |∆*H*_HA_(X)|. Among them, the difference between A and B is approximately 10 kcal/mol, and the difference of B and BH_2_ is approximately 14 kcal/mol.

Third, hydrogen affinity of A at position 2 |∆*H*_HA_(A_2_)| > hydrogen affinity of B at position 2 |∆*H*_HA_(B_2_)| >> hydrogen affinity of BH_2_ at position 2 |∆*H*_HA_(BH_22_)|, the presence of the unsaturated system increases the ability of carbonyl compounds to accept hydrogen atoms. If the reduction reaction starts with the transfer of hydrogen atoms, the carbonyl group is easier to be reduced than this group in the saturated system.

Fourth, the difference ∆∆*H*_HA_(B-BH_2_) of the hydrogen atom affinity of the carbonyl group is not far from the difference ∆∆*H*_H_-_A_ (B-BH_2_) of hydride affinity in B and AH_2_, which indicates that the effect of the conjugation system on the hydride affinity and hydrogen atom affinity of carbonyl groups is comparable, and the conjugation energy of the conjugated structure is approximately 15 kcal/mol.

Finally, the absolute value of the hydrogen atom affinity of the carbonyl group in the same compound is higher than that of the corresponding C=C bond, which is consistent with the trend of hydride affinity. The addition of hydrogen atoms to unsaturated bonds is similar to the addition of hydride ions. Hydrogen atoms are partially electronegative. However, because of their relatively small charge density, hydrogen atoms are less selective for reduction compared to hydride ions. Therefore, the difference in hydride affinity between the two types of unsaturated bonds in the same compound (∆∆*H*_H_-_A_(X_2_ − X_4_)) is slightly greater than their difference in hydrogen atom affinity (∆∆*H*_HA_(X_2_ − X_4_)).

### 3.3. Hydrogen Atom Affinity and Proton Affinity of Radical Anion Intermediate in α,β-Unsaturated Carbonyl Compounds

The experimental results in Table 2 show that the hydrogen atom affinity ∆*H*_HA_(X^•−^) of the radical anion intermediates in the three types of carbonyl compounds falls in the range of −45.26 to −67.39 kcal/mol. They are all large absolute values, making them good hydrogen atom acceptors.

The hydrogen atom affinity of the saturated ketone radical anion is very similar to the values of the other two types of compounds (A and B), indicating that the electrons activate the carbonyl group and weaken the π-bond, and the activation of the unsaturated system by electrons is less than that of the saturated system. A comparison of hydrogen atom affinity between the radical anions in position 2 and position 4 in the same compound indicates that the latter is still large in value, although its absolute value is reduced compared to the former. It can be seen that an electron obtained by the neutral compound is more likely to be added to the carbonyl group, and the radical anion formed is more inclined to accept a hydrogen atom to form a negative oxygen ion than a carbanion.

Table 2 shows that the proton affinity ∆*H*_PA_(X^•−^) of the radical anion is roughly in the range of −10.13 to −28.23 kcal/mol, where all radical anions are mild proton acceptors, as well as medium-strength Bronsted bases. For the same substituent, the proton affinity of radical anions in these compounds at three positions decreases in the following order: the proton affinity of compound A forming radical anions at position 2−|∆*H*_PA_ (A^2•−^)| > |∆*H*_PA_ (B^2•−^)| > |∆*H*_PA_(BH_2_^2•−^)| > |∆*H*_PA_ (A^4•−^)| > |∆*H*_PA_ (B^4•−^)|. The value of ||∆*H*_PA_ (A^2•−^)|−|∆*H*_PA_(AH_2_^2•−^)|| is approximately 3~5kcal/mol; it is not relevant to the substituent. The difference in proton affinity is also small between carbonyl radical anions, while the radical anions in position 2 of unsaturated carbonyl compounds have a slightly greater capability to accept protons than the radical anions in position 4.

Generally, the absolute value of hydrogen atom affinity in radical anion X^•−^ is approximately 38 kcal/mol greater than that of the corresponding proton affinity, which means that the radical anion is much more capable of accepting hydrogen atoms than protons under the same conditions. Therefore, for the electron transfer-initiated hydride reduction reactions of such compounds, electron–hydrogen atom (e^−^–H^•^) transfer is most likely subject to the multi-step hydride transfer mechanism.

### 3.4. Electron Affinity of α,β-Unsaturated Carbonyl Compounds and Its Radical Intermediate

#### 3.4.1. Single-Electron Reduction Potentials of α,β-Unsaturated Carbonyl Compounds

The data in Table 1 show that the single-electron reduction potentials of these three types of carbonyl compounds are negative and large in value between −1.771 and −2.595 (V vs. Fc^+/0^). Because these compounds are extremely weak single-electron acceptors, it is difficult for electron-initiated hydride transfer to occur.

For the same substituent, the reduction potentials of A and B are very close, and their values are both higher than that of BH_2_, indicating that the presence of the conjugated system enhances the ability of compounds to accept electrons, and newly added electrons can be dispersed in the conjugated system.

**Figure 5:**
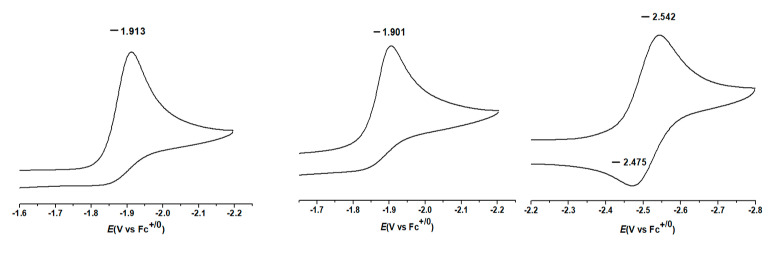
Cyclic voltammetry (CV) of A, B, and BH_2_ (R=CH_3_) in deaerated acetonitrile containing 0.1 M n-Bu_4_NPF_6_ as supporting electrolyte.

Both A and B are irreversible reduction potentials, while BH_2_ is a partially reversible reduction potential (Figure 5). The radical anion formed by unsaturated carbonyl compounds accepting one electron is unstable.

#### 3.4.2. Single-Electron Reduction Potentials of Radical Intermediate in α,β-Unsaturated Carbonyl Compounds 

The single-electron reduction potentials of a radical intermediate in carbonyl compounds (A, B and AH_2_) is obtained by detecting the oxidation potentials of the corresponding anions. Given two types of unsaturated bonds in unsaturated carbonyl compounds, corresponding carbon and oxygen radical intermediates can be generated. The reduction potentials of radical intermediates in these three types of carbonyl compounds are in the range of −0.486 to −0.787 (V vs. Fc^+/0^), indicating that the free radicals of these compounds are weak electron acceptors, and only partially stable to certain extent. Compared with BNAH (0.219 V) and HEH (0.479 V), they have a high electron-donating capacity and can be used as a single-electron reducing agent.

**Scheme 4:**
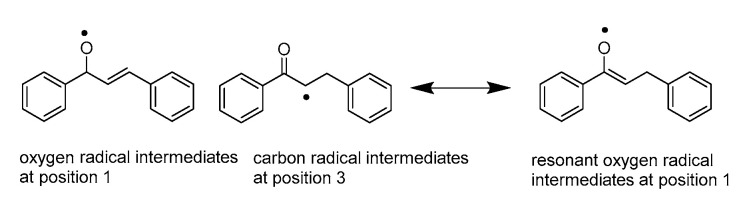
The free radical of an unsaturated carbonyl compound and its conjugate form.

The reduction potentials of unsaturated ketone radical intermediates is lower than that of corresponding saturated ketone radical intermediates, with a difference of more than 100 mV. The presence of the conjugated system stabilizes radical intermediates and reduces their electron-accepting capability. The reduction potentials of carbon radical intermediates at position 3 in the same compound is less than that of oxygen radical intermediates at position 1, e.g., *E*_red_(AH_2_) < *E*_red_(A´H_2_), which may be due to the fact that carbon radical intermediates at position 3 can form more stable “resonant” oxygen radical intermediates (Scheme 4), while oxygen radical intermediates at position 1 cannot resonate with the benzene ring double bond due to the blocking of the saturated carbon bond. The reduction potentials of A and B radical intermediates are very close to each other, indicating that the change in the position of the substituent has little effect on the reduction potentials of radical intermediates.

### 3.5. Establishment of Molecule ID Card for Carbonyl Compounds

Can the chemical properties of organic molecules and reaction intermediates be shown on a single spectrum? To this end, in 2010, we proposed the concept of the “Molecule ID Card” for organic compounds [31]; it contains the thermodynamic and electrochemical data related to the compound, through which we can visually and quantitatively predict the chemical properties of the organic compounds and its reaction intermediates, such as its pH value, electrophilicity, nucleophilicity, and redox capacity. We can also predict the reaction process and possible products and resolve the reaction mechanism for the purpose of designing a variety of chemical reactions.

**Figure 6:**
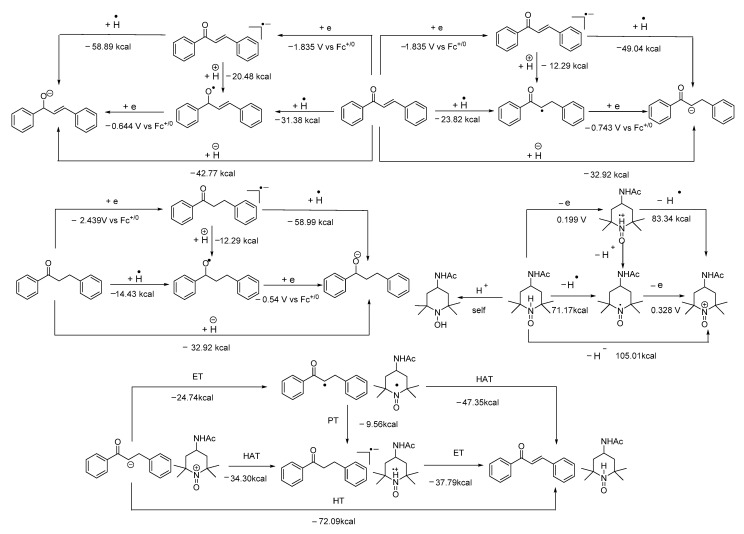
“Molecule ID Card” of ketones and Ac-tempo^+^ and thermodynamic Characteristic Graph (TCG) of the hydride transfer reaction.

Based on the abovementioned four thermodynamic parameters ∆*H*_H_−_A_(X), ∆*H*_HA_(X), ∆*H*_HA_(X^•−^) and ∆*H*_PA_(X^•−^), and the electrochemical parameters *E*_red_(X) and *E*_red_(XH), we created our own “Molecule ID Cards” for the three studied systems (Figure 6). The reaction processes of all reactants can also be studied according to the “Molecule ID Cards”. For the thermodynamic determination herein (Supporting Information), we studied the corresponding hydride transfer process using carbonyl compounds as hydride donors and Ac-tempo^+^ as hydride acceptors.

We can make the following inferences about the reaction processes and mechanisms of these two types of compounds:

(1) The mixture (if any) of carbonyl hydride ions and Ac-tempo^+^ will be rapidly subject to hydride transfer to produce corresponding α,β-unsaturated carbonyl compounds and Ac-tempoH, as shown in the conducted experiments. A comparison of the energies between one-step hydride transfer and multi-step hydride transfer initiated by electrons or hydrogen atoms indicates that these three pathways are energetically favorable, and one-step hydride transfer has the greatest thermodynamic driving force;
(2) When two types of radicals come in contact, either hydrogen–atom transfer or proton transfer may occur. Electron transfer is energetically analyzed as forbidden, and hydrogen atom transfer is significantly better than proton transfer;
(3) When the radical anions and radical cations of two types of compounds meet, it is difficult for them to be inclined to hydrogen atom transfer or proton transfer but very easy for them to be stimulated to electron transfer, because the first two types of transfer are energetically forbidden, while electron transfer is a spontaneous process wherein these two types of intermediates can achieve a stable electronic structure;
(4) When neutral α,β-unsaturated carbonyl compounds and Ac-tempoH are mixed, based on TCG analysis, the transfer in all steps is forbidden.

## 4. Conclusions

In this paper, a series of three carbonyl compounds and their corresponding reduction products were synthesized [29,30,32,33,34]. The reduction potentials of carbonyl compounds and their corresponding radical intermediates were determined by two electrochemical techniques [35], namely CV and OSWV, and the hydride affinity of unsaturated bonds in two types of α,β-unsaturated carbonyl compounds and one type of saturated carbonyl compounds were determined by ITC [36]. We further calculated the hydrogen atom affinity of X and the hydrogen atom affinity and proton affinity of reaction intermediates based on thermodynamic cycles.

The following conclusions were drawn:

(1) All three series compounds are weak hydride acceptors. The ability of unsaturated carbonyl compounds to accept hydride is better than that of saturated carbonyl compounds. All three series compounds are extremely weak hydrogen atom acceptors. Hydride transfer initiated by hydrogen atom transfer is not the best transfer path. Such compounds are extremely weak single-electron acceptors. Their corresponding anions X^−^ are all extremely strong single-electron reducing agents. The radical anion X^•−^ of carbonyl compounds is a strong hydrogen atom acceptor and weak proton acceptor, which is difficult to stabilize.
(2) A comparison of hydride affinity and hydrogen atom affinity between two types of unsaturated bonds in α,β-unsaturated carbonyl compounds indicates that the absolute value of them for carbonyl groups is slightly larger than that of carbon–carbon double bonds. The selective reduction of them is more difficult to control. However, the presence of a conjugated system activates carbonyl groups and enhances the capability of carbonyl groups to accept hydride ions and hydrogen atoms.
(3) We constructed “Molecule ID Cards” for carbonyl compounds by means of thermodynamic and electrochemical data to not only quantitatively determine the chemical properties of carbonyl compounds and their reaction intermediates but also easily predict the reaction mechanism.

## Figures and Tables

**Table 1:** Reaction Enthalpy Changes of the Unsaturated Three Carbonyl Compounds in Acetonitrile at 298 K (kcal/mol) and Reduction Potentials (V vs Fc^+/0^) of A, B, A’H_2_, B’H_2_, AH_2_, BH_2_ in Acetonitrile at 298 K.

Substituent	∆*H*_rxn_^a^				*E*_red_^b^			
	∆*H*_H-A_^2^	∆*H*_H-A_^4^	CV	OSWV	CV	OSWV	CV	OSWV
A			*E*_red_(A)		*E*_red_(A′H_2_)		*E*_red_(AH_2_)	
CH_3_O	−70.68	−75.35	−1.920	−1.899	−0.672	−0.700	−0.740	−0.787
CH_3_	−65.26	−73.36	−1.913	−1.889	−0.660	−0.680	−0.726	−0.763
H	−62.24	−72.09	−1.876	−1.835	−0.609	−0.644	−0.690	−0.743
Cl	−52.27	−64.93	−1.798	−1.771	−0.587	−0.612	−0.671	−0.719
B			*E*_red_(B)		*E*_red_(B′H_2_)		*E*_red_(BH_2_) ^c^	
CH_3_O	−72.65	−77.35	−1.929	−1.904	−0.651	−0.692	−0.723	−0.781
CH_3_	−66.01	−74.64	−1.901	−1.876	−0.643	−0.672	−0.714	−0.761
H	−62.24	−72.09	−1.876	−1.835	−0.609	−0.644	−0.690	−0.743
Cl	−53.27	−69.33	−1.811	−1.784	−0.577	−0.608	−0.682	−0.717
BH_2_			*E*′_red_(BH_2_) ^c^		*E*_red_(BH_4_)			
CH_3_O	−86.24		−2.586	−2.595	−0.500	−0.538		
CH_3_	−81.55		−2.509	−2.515	−0.489	−0.526		
H	−76.10		−2.437	−2.439	−0.478	−0.510		
Cl	−64.90		−2.244	−2.215	−0.457	−0.486		

^a^ ∆*H*rxn is obtained based on the reaction heats calculated using Equation (5). The unit is kcal/mol. The experimental error is 0.5 kcal/mol. ^b^ The unit in volts vs Fc^+/0^ and reproducible to 5 mV (the experimental error) or better. ^c^ They are all derived from compound BH_2_.

**Table 2:** Hydride and Hydrogen Affinities of Unsaturated Ketones (A, B) and Saturated Ketones (BH_2_), Hydrogen and Proton Affinities of X^•−^ in Acetonitrile.

Position	2	4	2	4	2	4	2	4
Substituent	∆*H*_H_-_A_(X) ^a^		∆*H*_HA_(X) ^b^		∆*H*_HA_(X^•−^) ^b^		∆*H*_PA_(X^•−^) ^b^	
A								
CH_3_O	−34.33	−29.66	−24.24	−21.58	−51.93	−47.26	−14.81	−12.15
CH_3_	−39.75	−31.65	−29.19	−23.01	−57.12	−49.02	−19.54	−13.35
H	−42.77	−32.92	−31.38	−23.82	−58.89	−49.04	−20.48	−12.92
Cl	−52.74	−40.08	−40.61	−30.42	−67.39	−54.73	−28.23	−18.04
B								
CH_3_O	−32.36	−27.66	−22.08	−19.44	−50.08	−45.38	−12.77	−10.13
CH_3_	−39.00	−30.37	−28.26	−21.68	−56.07	−47.44	−18.30	−11.73
H	−42.77	−32.92	−31.38	−23.82	−58.89	−49.04	−20.48	−12.92
Cl	−51.74	−35.68	−39.52	−25.98	−66.69	−50.63	−27.44	−13.90
BH_2_								
CH_3_O	−18.77		−4.93		−52.45	3.22	−11.59	−14.81
CH_3_	−23.46		−9.35		−55.29	5.39	−14.15	−19.54
H	−28.91		−14.43		−58.99	3.00	−17.48	−20.48
Cl	−40.11		−25.07		−65.01	5.28	−22.95	−28.23

^a^ ∆*H*_H_-_A_(X) values of unsaturated and saturated ketones were estimated from Equation (5). ^b^ ∆*H*_HA_(X), ∆*H*_HA_(X^•−^), and ∆*H*_PA_(X^•−^) were estimated from Equations (6)~(8), respectively, and choosing the reduction potentials of X and XH^•^ detected by OSWV method (Table 1) since the values from OSWV were identified to be closer to the corresponding standard redox potentials than the values from CV.

## Data Availability

Not applicable.

## Supplementary Materials

The following supporting information can be downloaded at: https://www.mdpi.com/article/10.3390/molecules28062862/s1. Detailed 1H NMR and 13C NMR data of typical compounds are shown in SI.
