# Supplementary material for: Thermodynamics Evaluation of Selective Hydride Reduction for α,β-Unsaturated Carbonyl Compounds
Source: Molecules. 2023 Mar 22;28(6):2862. doi: 10.3390/molecules28062862 (PMC10051270; doi:10.3390/molecules28062862)
Supplement: Supplementary file 1 [file molecules-28-02862-s001.zip › molecules-2263034-supplementary.pdf]

Supporting Information for

Thermodynamics Evaluation of Selective Hydride Reduction for α, β-Unsaturated Carbonyl Compounds

Bao-Long Chen*, Sha Jing and Xiao-Qing Zhu
The State Key Laboratory of Elemento-Organic Chemistry, Collaborative Innovation Center of Chemical Science and Engineering , College of Chemistry, Nankai University, Tianjin 300071, P. R. China.
*E-mail: cblnku@163.com

Table of Contents

**SI.**	**Materials.**	**S2**
**SII.**	**Measurment of Redox Potentials.**	**S2-S3**
**SIII.**	**Isothermal Titration Calorimetry (ITC).**	**S3**
**SIV.**	**^{1}H-NMR and ^{13}C-NMR spectra of representative compounds.**	**S3-S25**
**SV.**	**Electrochemical spectra of representative compounds.**	**S26-S28**
**SVI.**	**ITC spectra of representative reactions.**	**S28-329**

SI.Materials.

Solvents and reagents were obtained from commercial sources and used as received unless otherwise noted. Reagent grade acetonitrile was refluxed over $KMnO_4$ and K_2CO_3 for at least eight hours and doubly distilled over P_2O_5 under argon and stored in the glove-box before use. All compounds were synthesized (Figure S1) according to literature methods. These references are shown in the original article.

The parent compound for donating hydride anions BH_2（R=Cl） (0.05mmol) was dissolved in 5 mL anhydrous anoxic acetonitrile in a glove box with high purity argon gas atmosphere and then a small amount of solid NaH (0.045mmol) powder was added into the mixture. The mixture was stirred at room temperature for 30 minutes and then filtered for use. Figure S2 shows the corresponding anion ^{1}HNMR analysis spectrum.

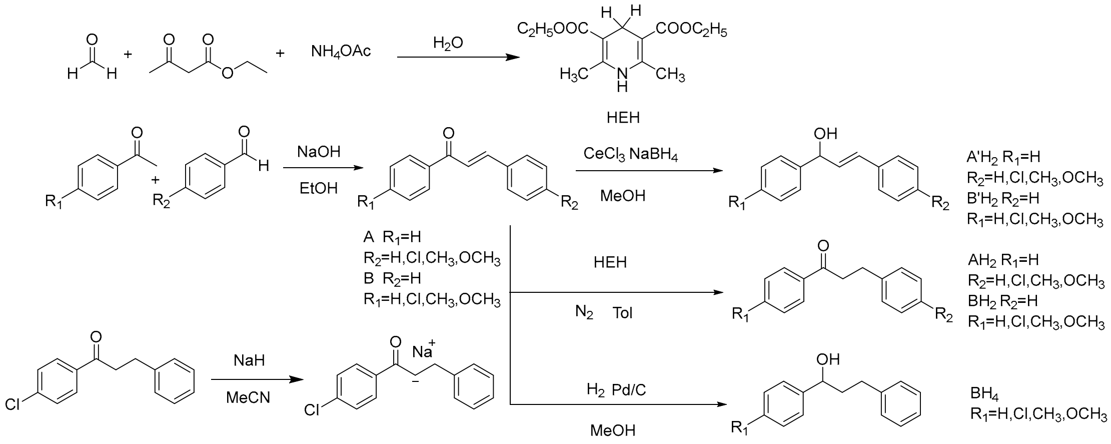

Figure S1. The synthesis route for all compounds

SII.Measurment of Redox Potentials.

The electrochemical experiments were carried out by cyclic voltammetry (CV) and osteryoung square wave voltammetry (OSWV) using BAS100B electrochemical apparatus in deaered acetonitrile under argon atmosphere at 298 K as described previously nBu_4NPF_6 (0.1M) in acetonitrile was employed as the supporting electrolyte. A standard three-electrode cell consists of a glassy carbon disk as work electrode, a platinum wire as a counter electrode, and 0.1 M $AgNO_3$/Ag (in 0.1 M n-Bu_4NPF_6- acetonitrile) as reference electrode. The ferrocenium/ferrocene redox couple ($Fc^{+/0}$) was taken as the internal standard. The reproducibilities of the potentials were usually ≤5 mV for ionic species and ≤10 mV for neutral species.

During the measure procedure, the compounds A, AH_2, A'H_2 will be solved in acetonitrile containing nBu_4NPF_6 (0.1M) All the same are true for B. In Table 1，E_{red}(BH_2) stands for the peak approach to positive direction，E'_{red}(BH_2) stands for the peak approach to negative direction.

In Table 1 on original article, E_{red}(A) is the potentials of compound A, E_{red}(A′H_2) is the potentials of A'H_2，E_{red}(AH_2) is the potentials of compound AH_2，the unit in volts vs $Fc^{+/0}$. All the same are true for B. But，the compound BH_2（R=Cl）is an exception，E_{red}(BH_2 R=Cl) is the potentials of compound BH_2(R=Cl) sodium salt.

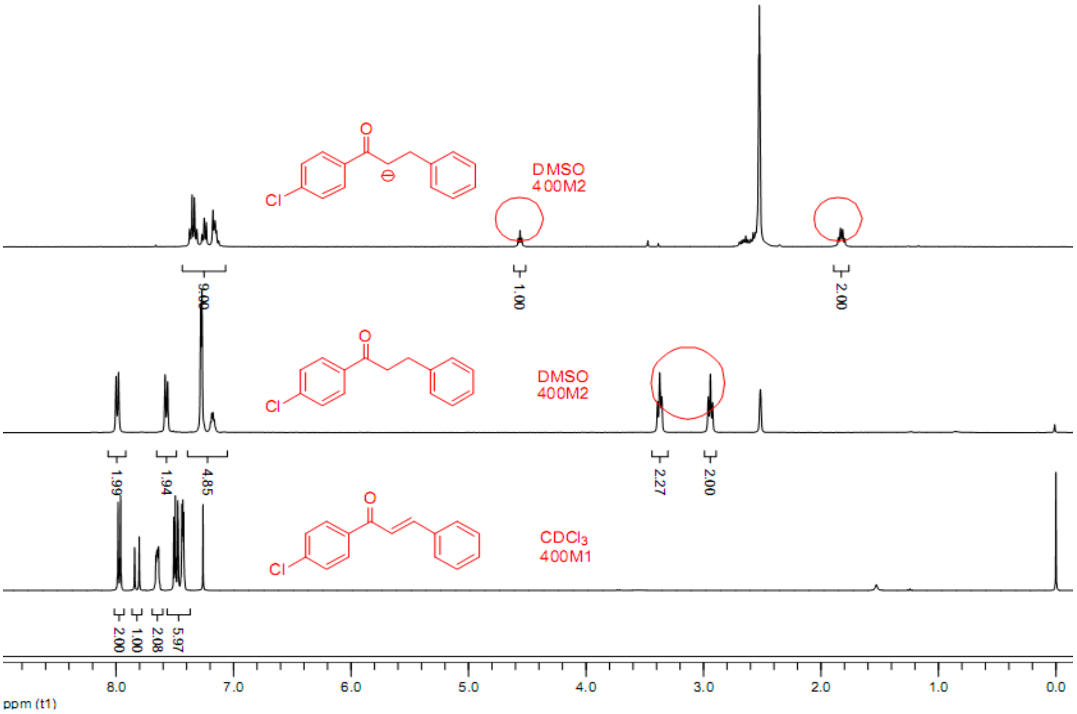

Figure S2. Comparison the ^{1}HNMR spectrum of the B, BH_2, B (R=Cl) sodium salt.

SIII.Isothermal Titration Calorimetry (ITC).

The titration experiments were performed on a CSC4200 isothermal titration calorimeter in acetonitrile at 298 K as described previously. The performance of the calorimeter was checked by measuring the standard heat of neutralization of an aqueous solution of sodium hydroxide with a standard aqueous HCl solution，the solvents used in the experiment were anhydrous and anaerobic acetonitrile. Ac-tempo$^+ClO_4^-$(1 mM) was used as the titration solution, 1 mL carbonyl compound anion XH was used as the reaction solution. The experiment was determined at 298K, the balance time is 400 s and the titration time is 400 s. The heat of reaction obtained by integrating the area of each peak (except the first peak) in the titration curve. The test was repeated at least 5 times for each sample, and the reaction heat error was ≤±0.5 kcal/mol. Note: typically the first injection shows less heat than expected. This is often due to diffusion across the tip of the needle or to difficulties in positioning the buret drive.

In Table 2 on original article, ΔH_{rxn4} of A was derived from the reaction of AH_2 and Ac-tempo$^+$, ΔH_{rxn2} of A was derived from the reaction of A'H_2 and Ac-tempo$^+$. All the same were for B. But，the compound BH_2（R=Cl）is an exception，ΔH_{rxn2} of BH_2 (R=Cl) was derived from the reaction of BH_2 (R=Cl) sodium salt and Ac-tempo$^+$.

ΔH_{rxn2} of BH_2 was derived from the reaction of BH_4 and Ac-tempo$^+$.

SIV. ^{1}H-NMR and ^{13}C-NMR spectra of representative compounds.

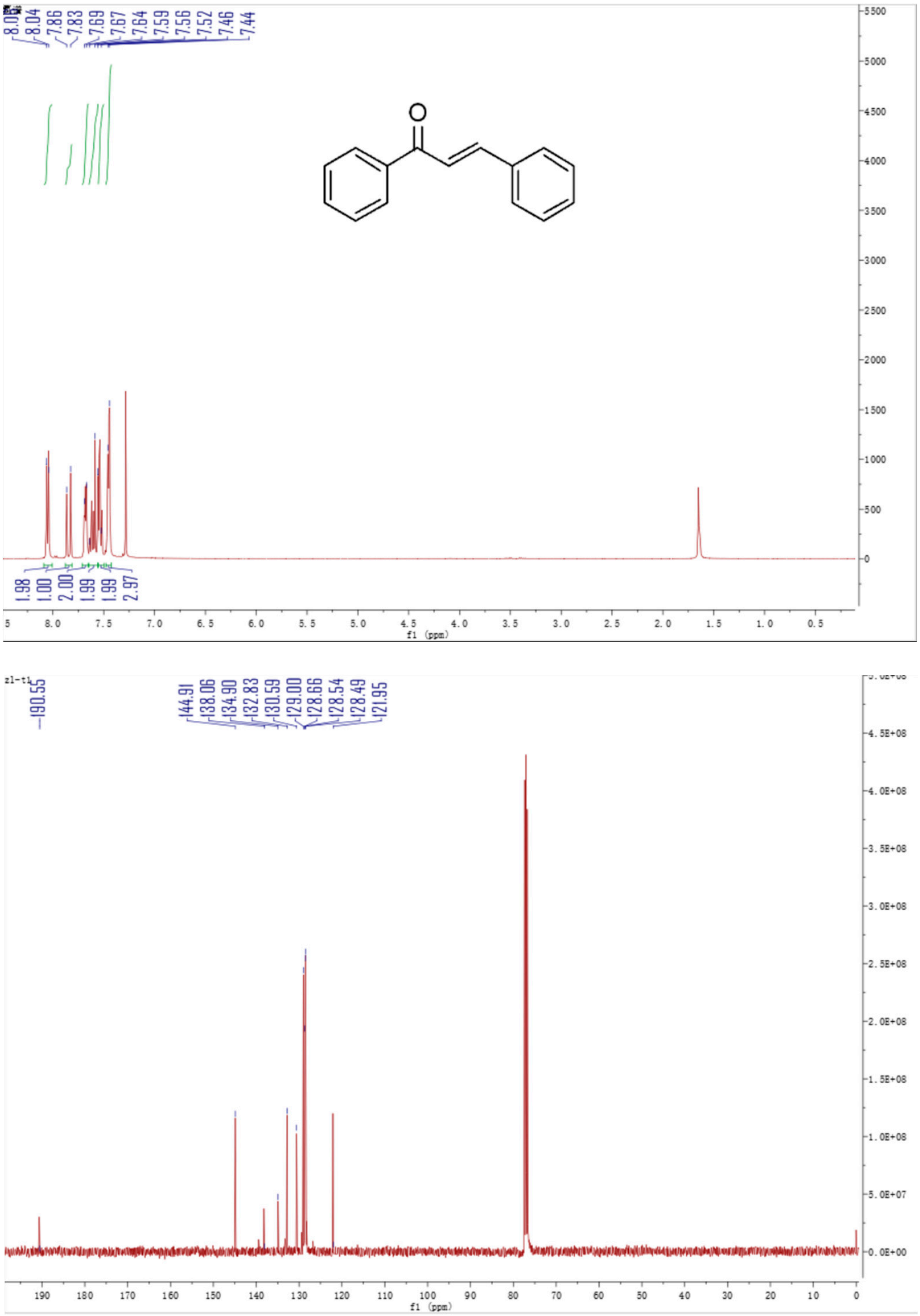

Scheme S1. ^{1}H-NMR and ^{13}C-NMR spectra of A (R=H).

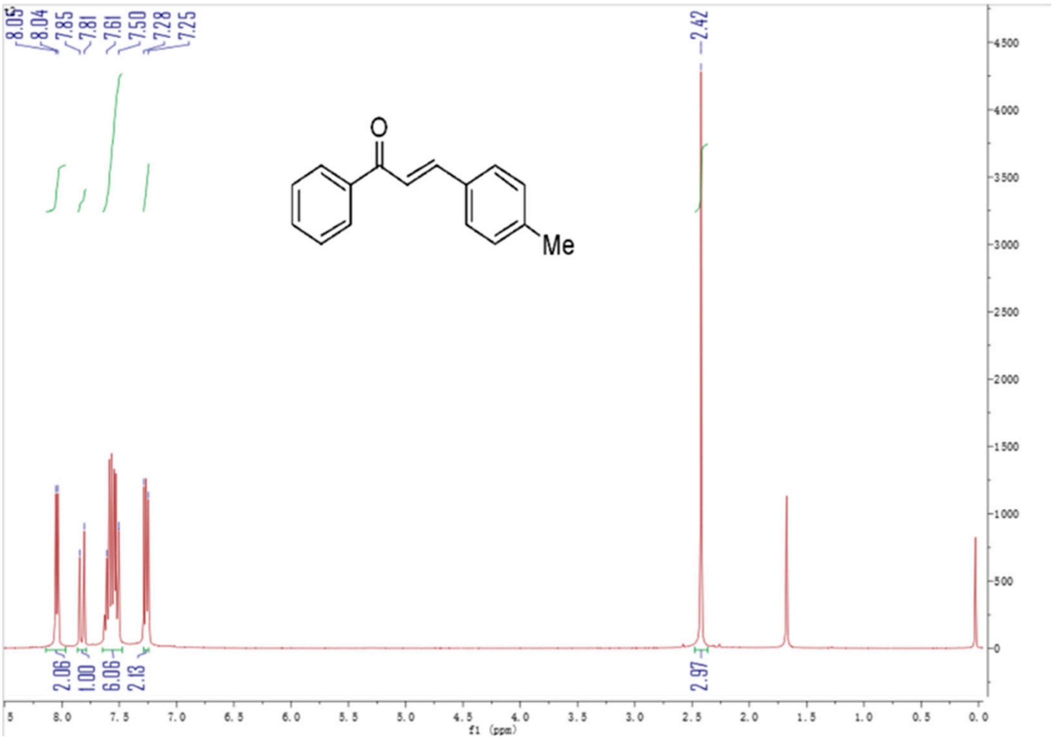

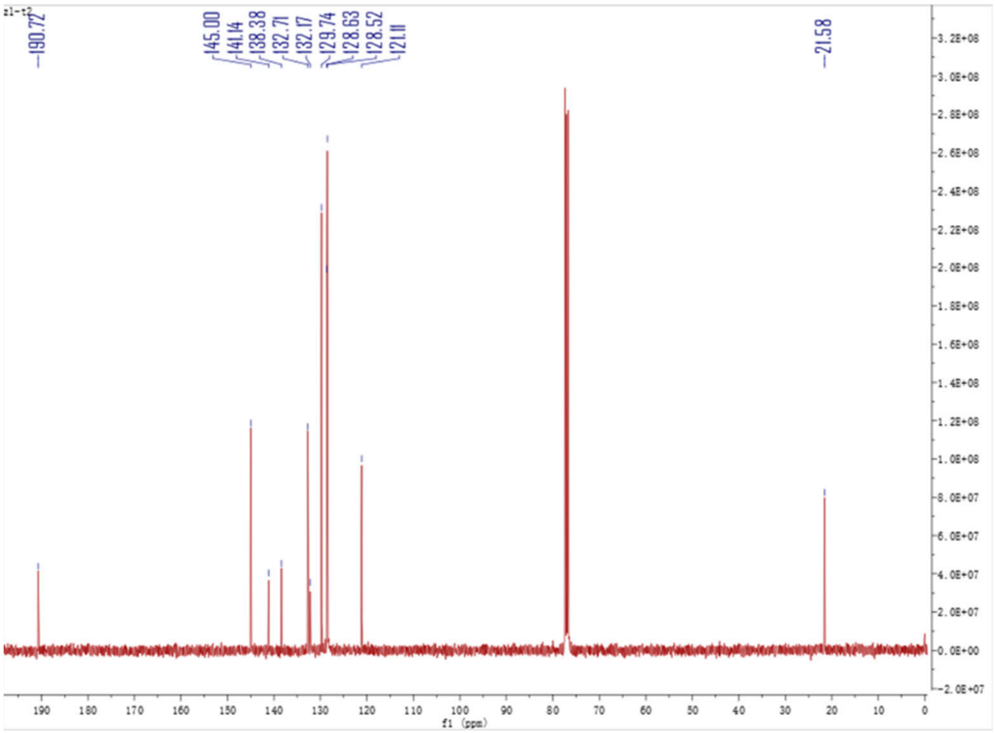

Scheme S2. ^{1}H-NMR and ^{13}C-NMR spectra of A (R=Me).

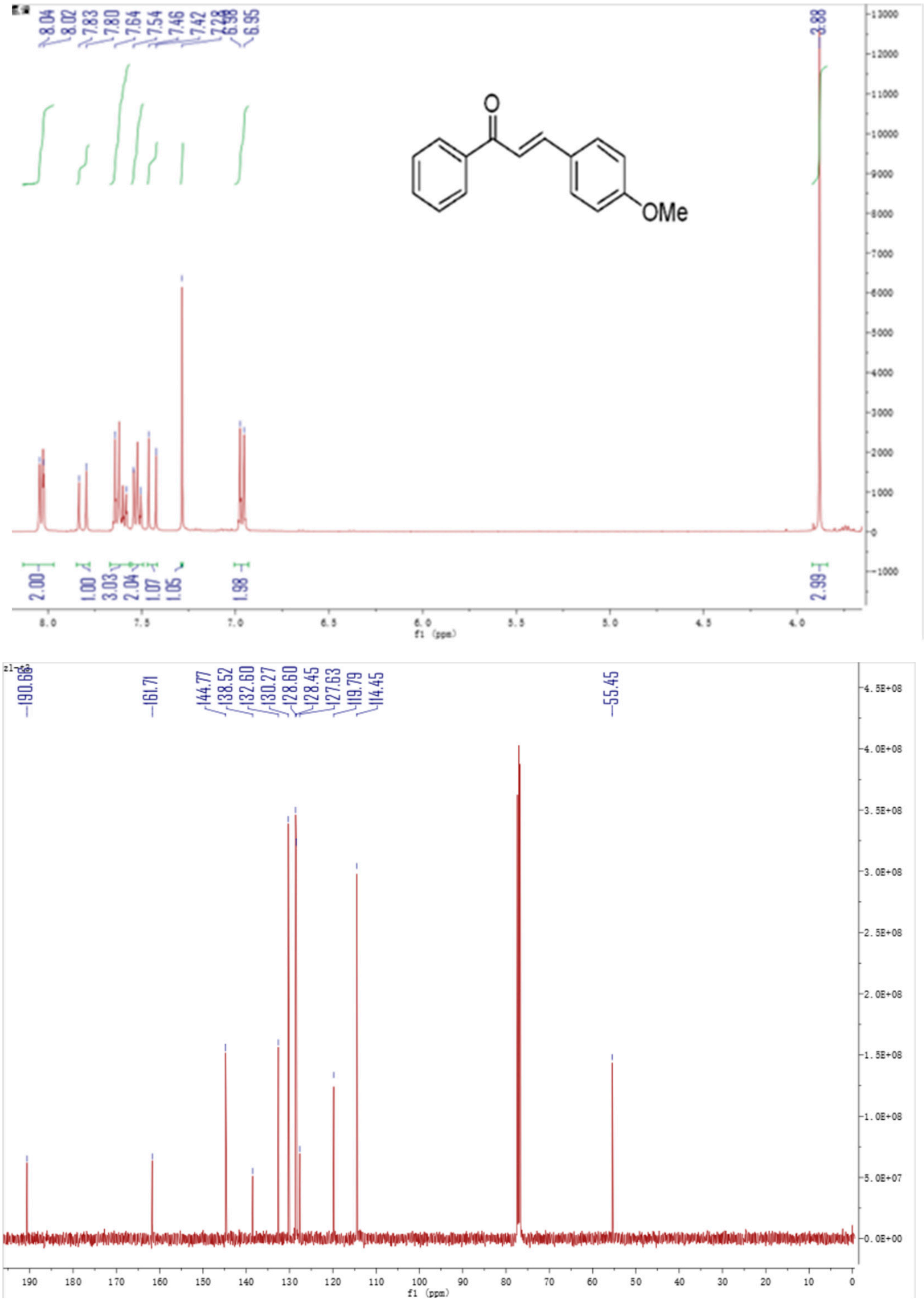

Scheme S3. ^{1}H-NMR and ^{13}C-NMR spectra of A (R=OMe).

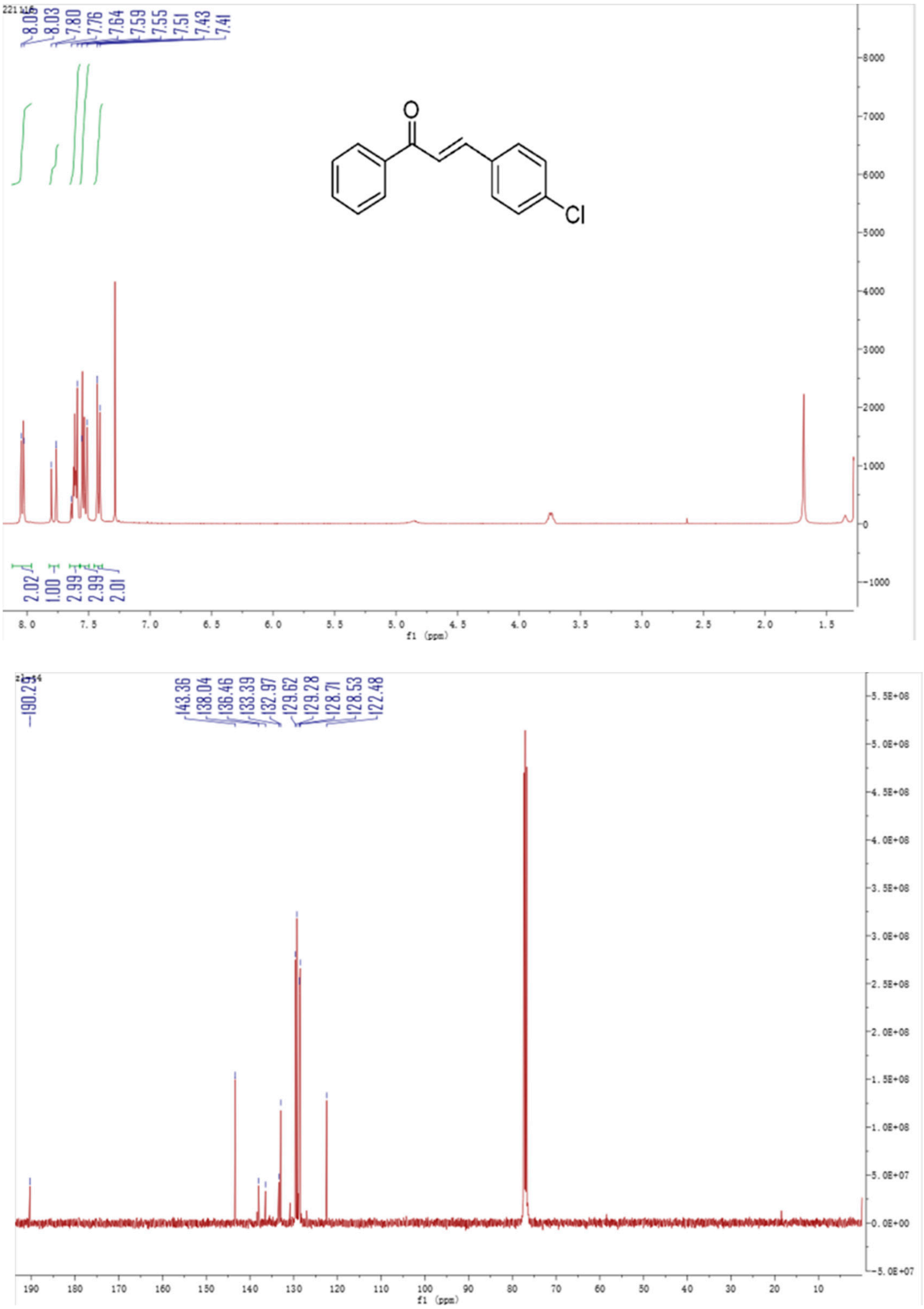

Scheme S4. ^{1}H-NMR and ^{13}C-NMR spectra of A (R=Cl).

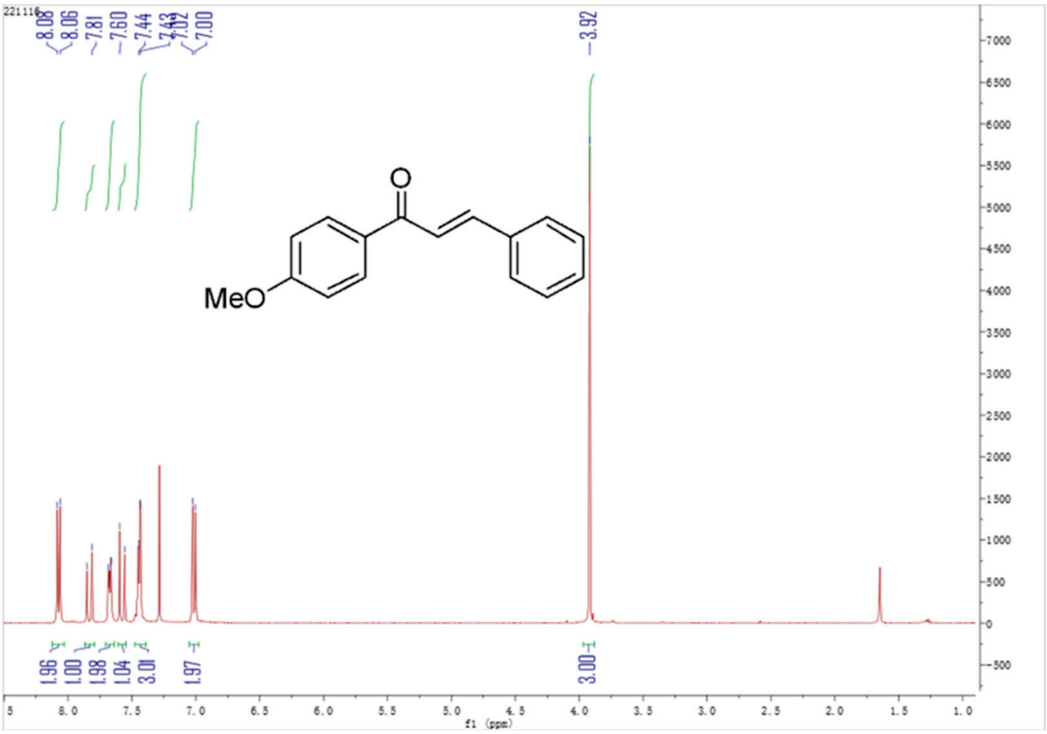

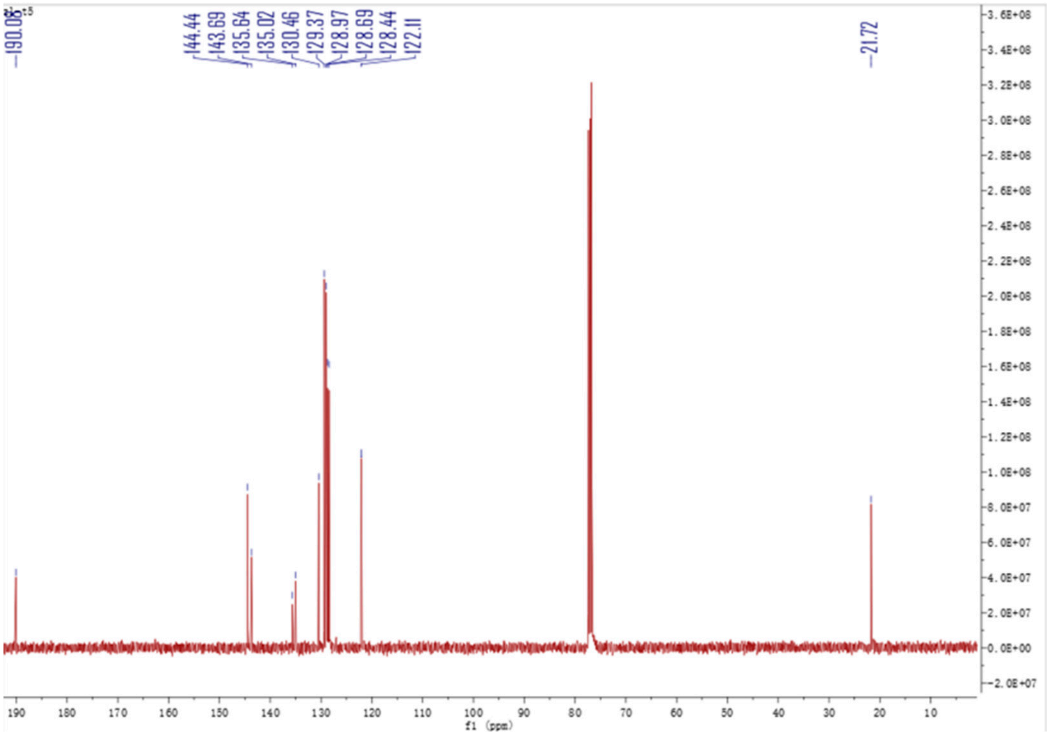

Scheme S5. ^{1}H-NMR and ^{13}C-NMR spectra of B (R=OMe).

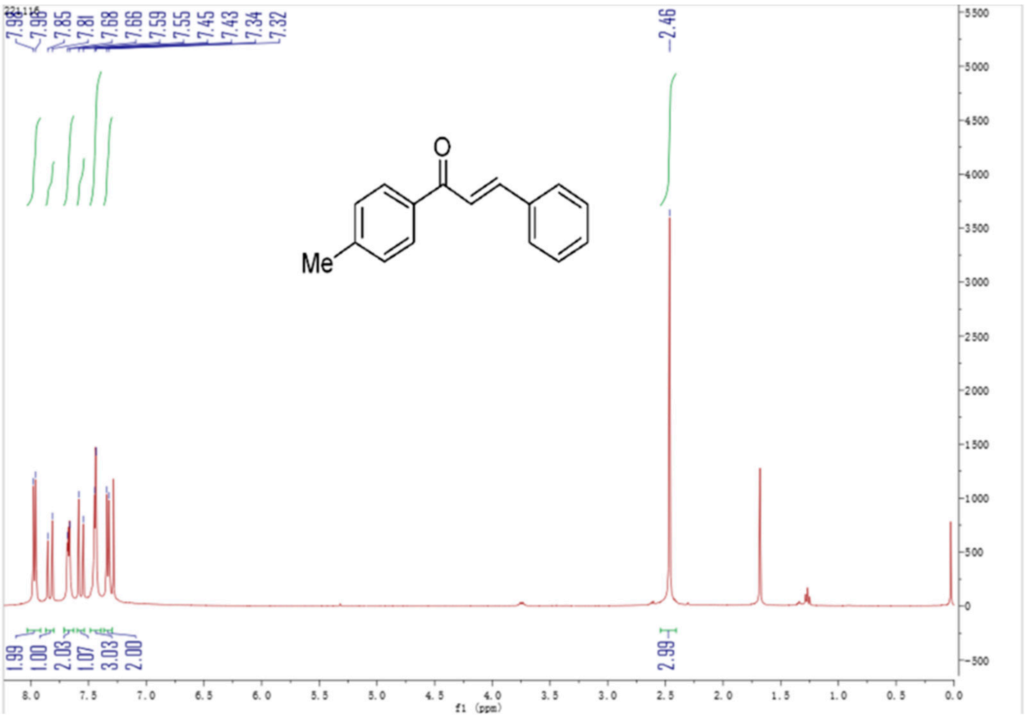

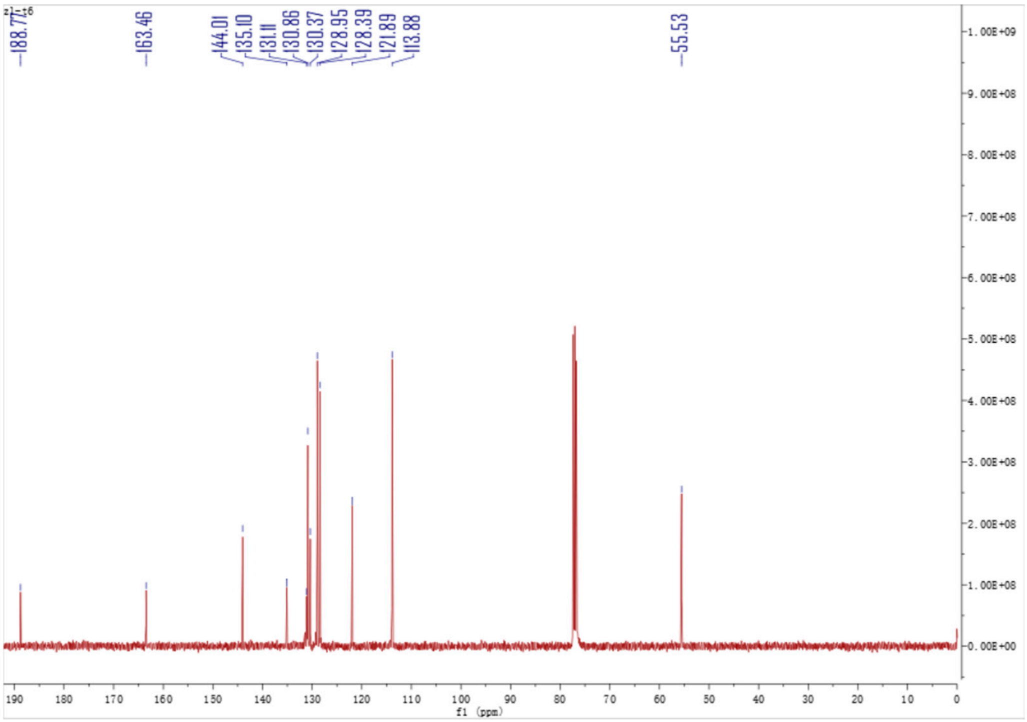

Scheme S6. ^{1}H-NMR and ^{13}C-NMR spectra of B (R=Me).

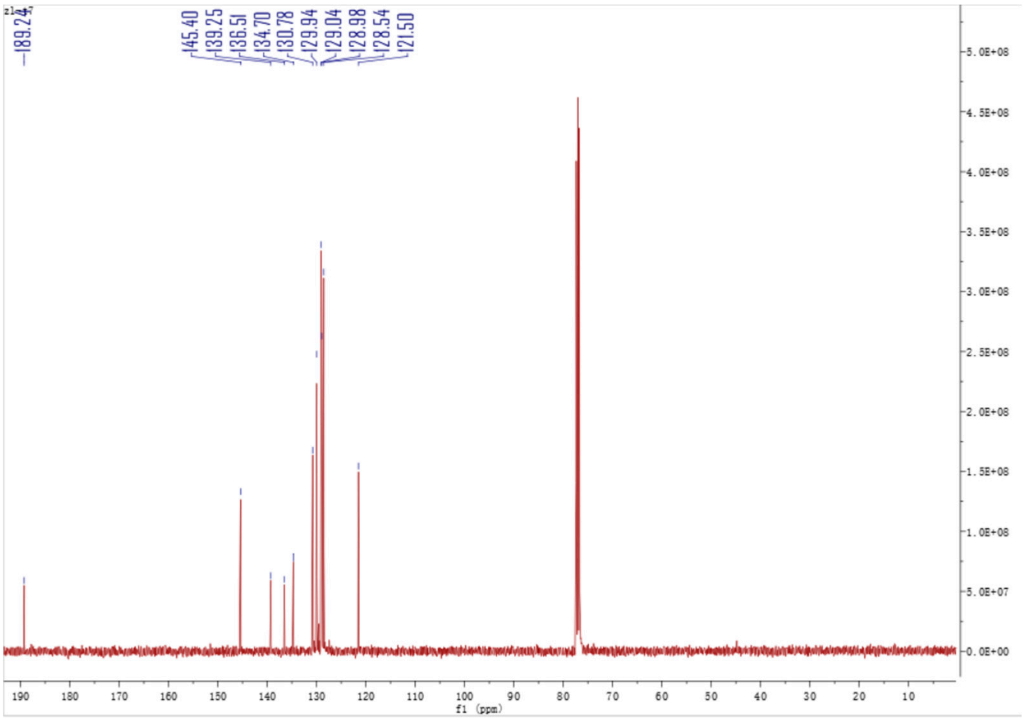

Scheme S7. ^{13}C-NMR spectra of B (R=Cl).

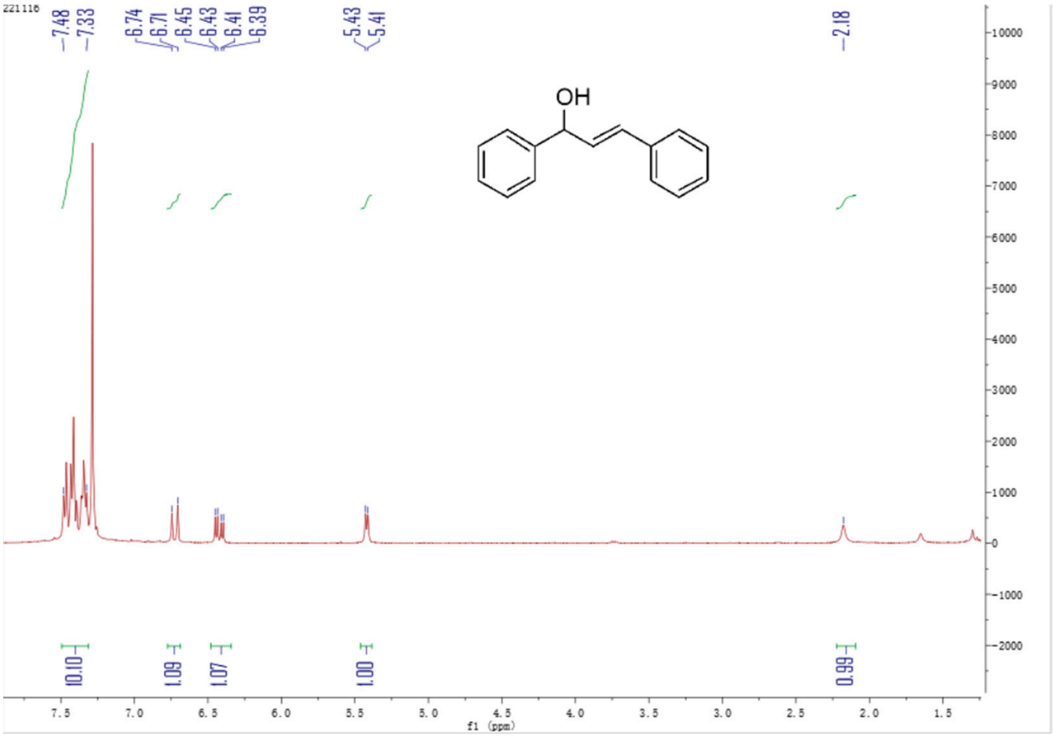

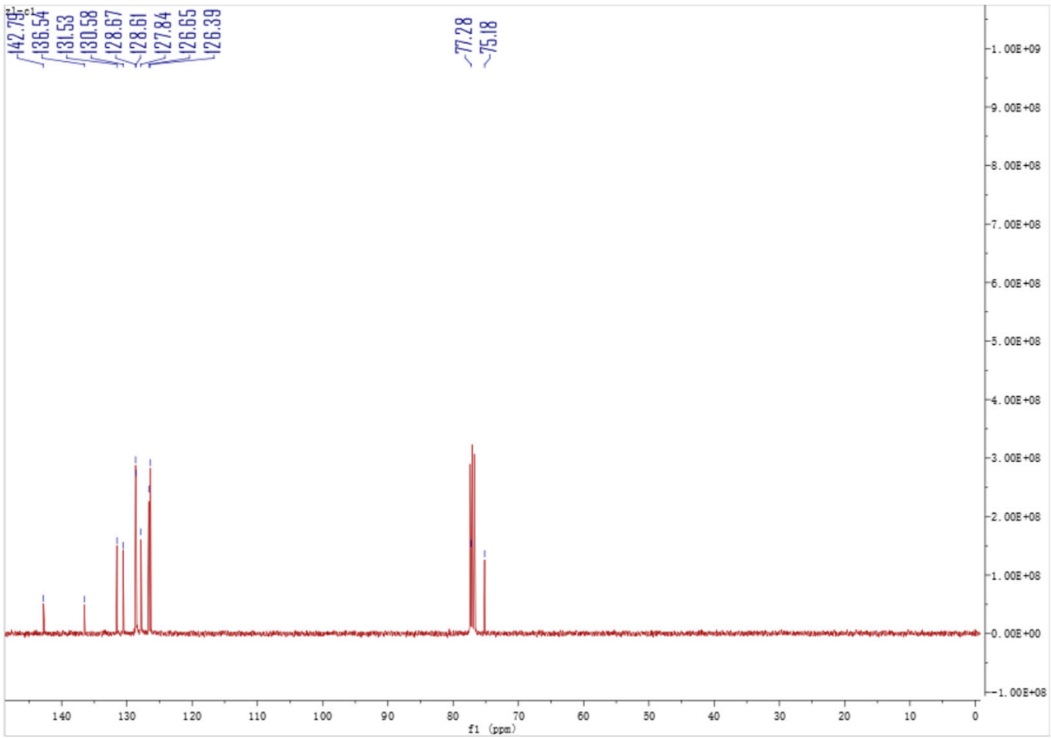

Scheme S8. ^{1}H-NMR and ^{13}C-NMR spectra of A'H_2 (R=H).

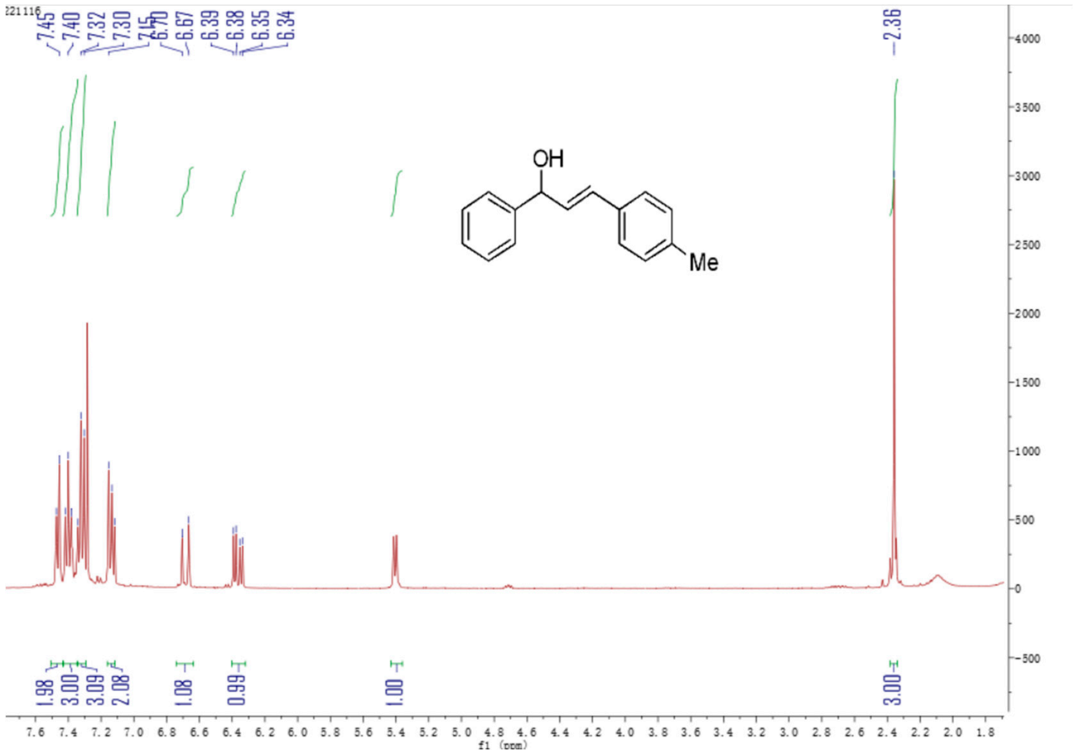

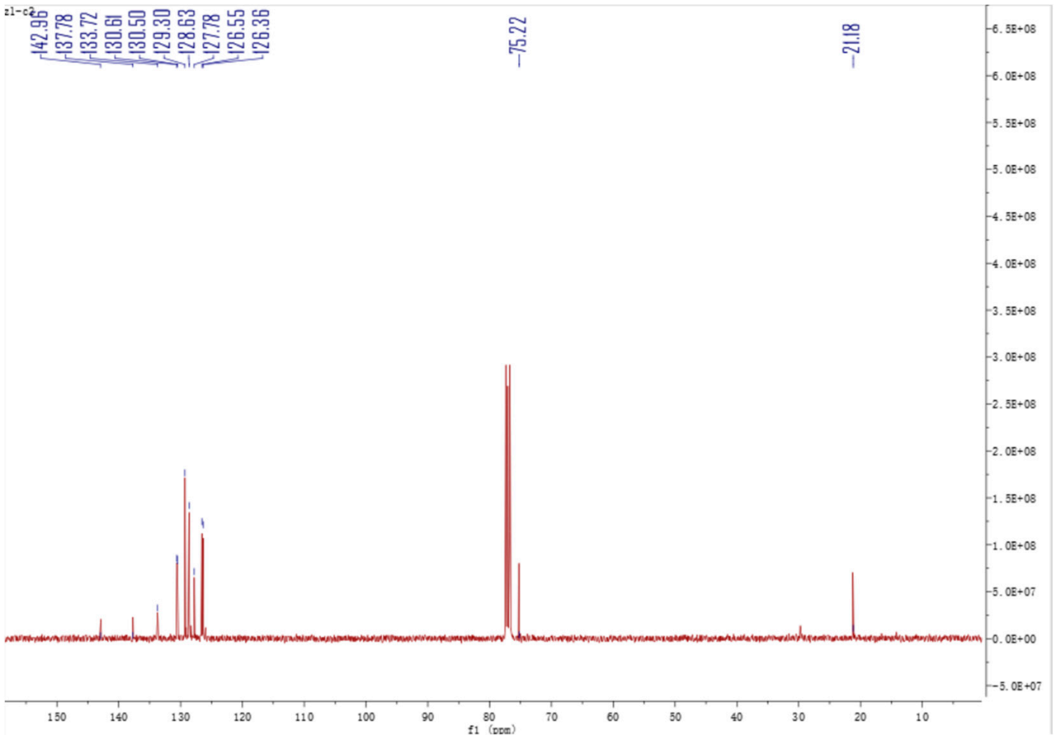

Scheme S9. ^{1}H-NMR and ^{13}C-NMR spectra of A'H_2 (R=Me).

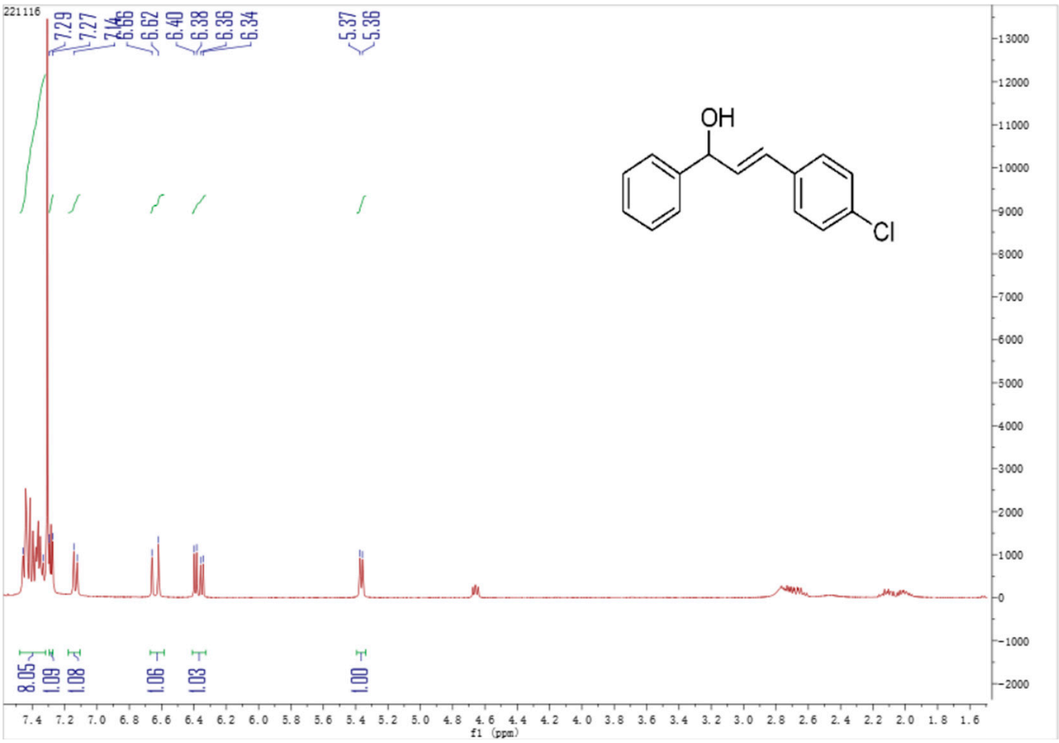

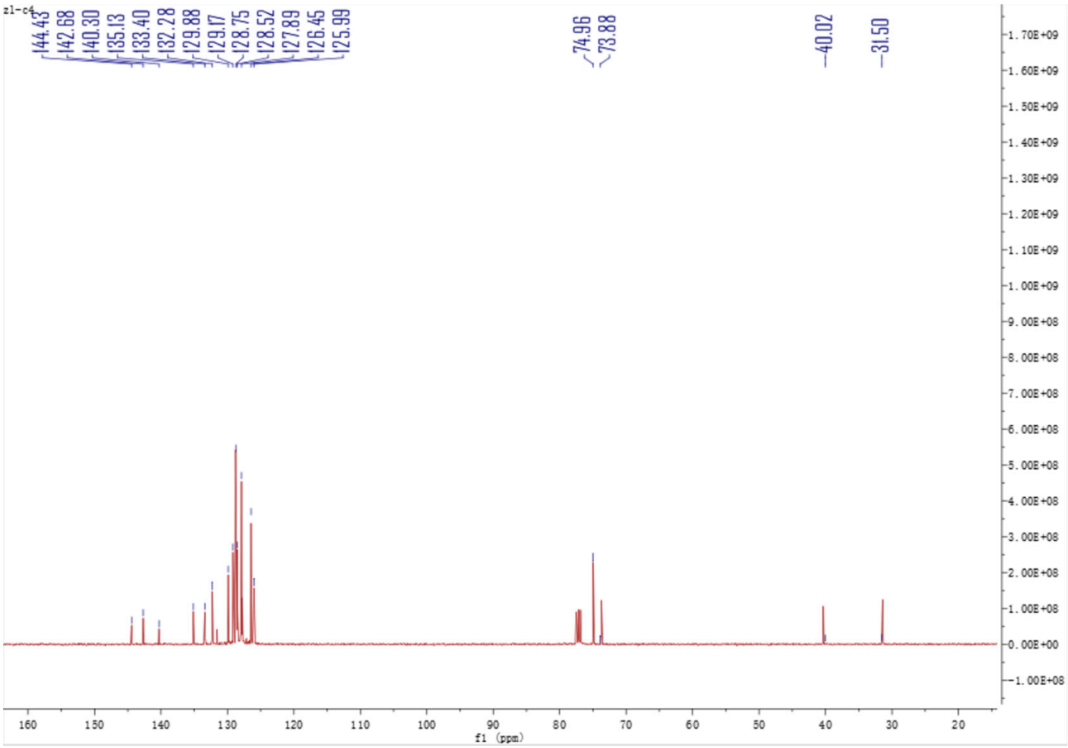

Scheme S10. ^{1}H-NMR and ^{13}C-NMR spectra of A'H_2 (R=Cl).

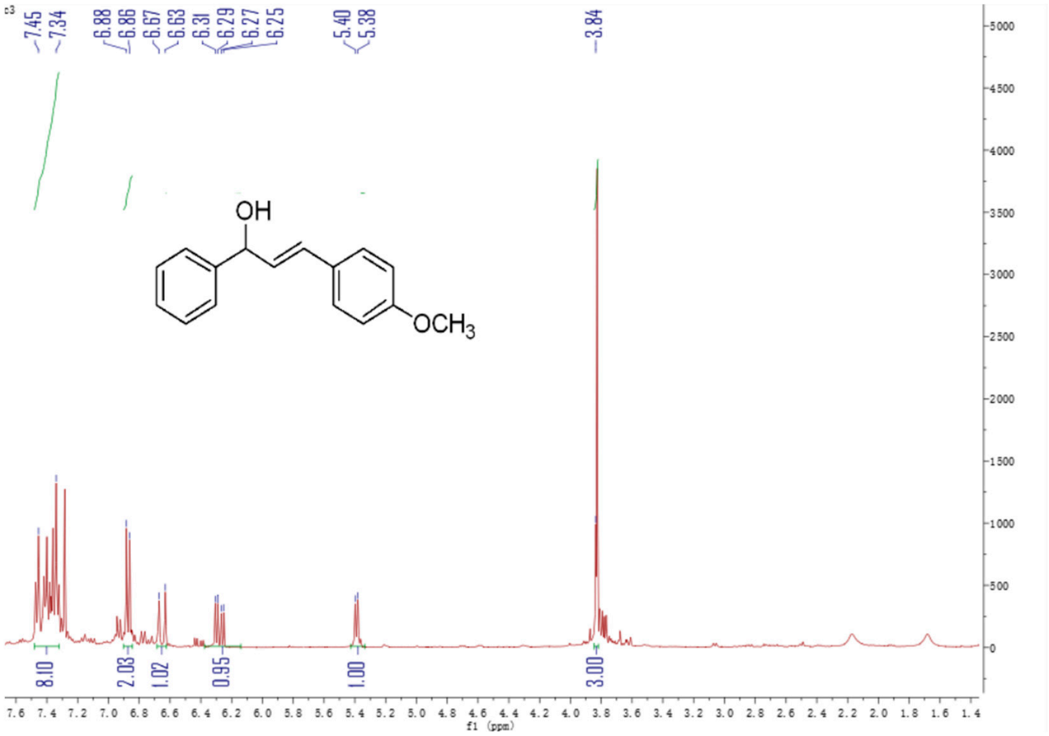

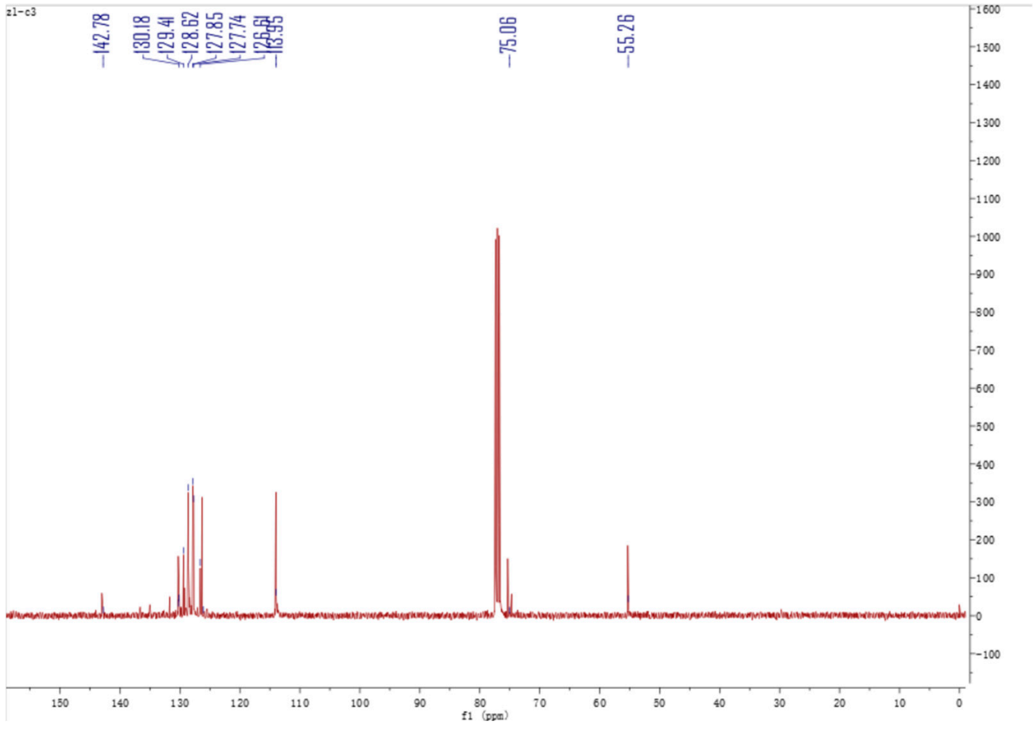

Scheme S11. ^{1}H-NMR and ^{13}C-NMR spectra of A'H_2 (R=OMe).

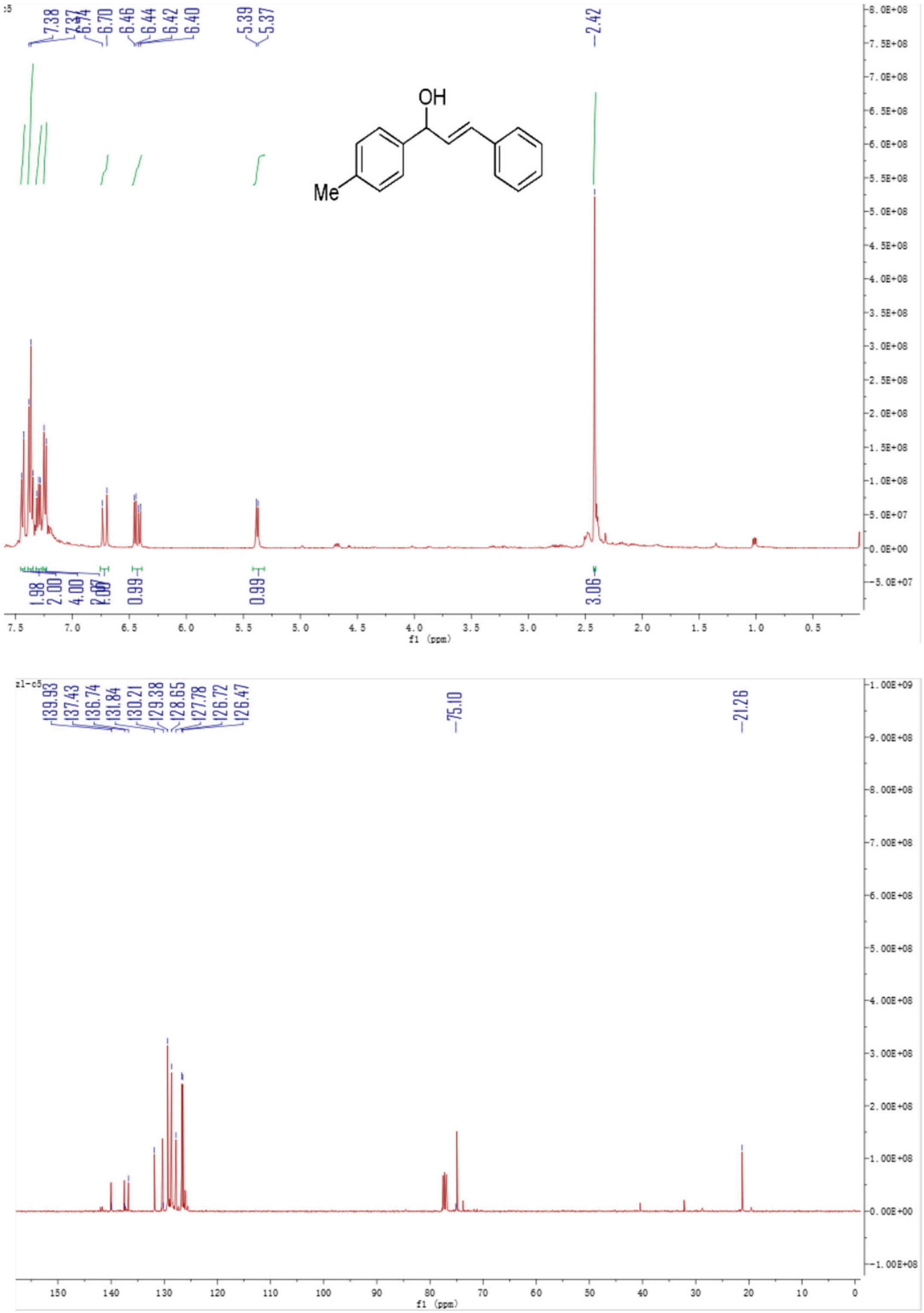

Scheme S12. ^{1}H-NMR and ^{13}C-NMR spectra of $B'H_2$ (R=Me).

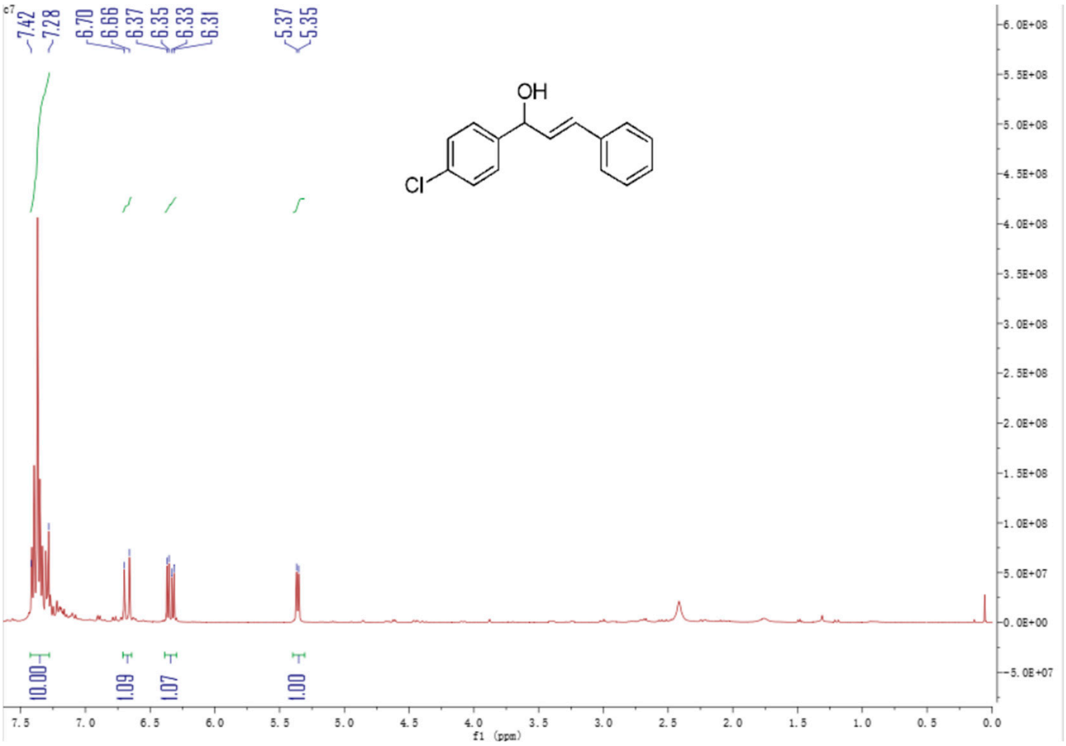

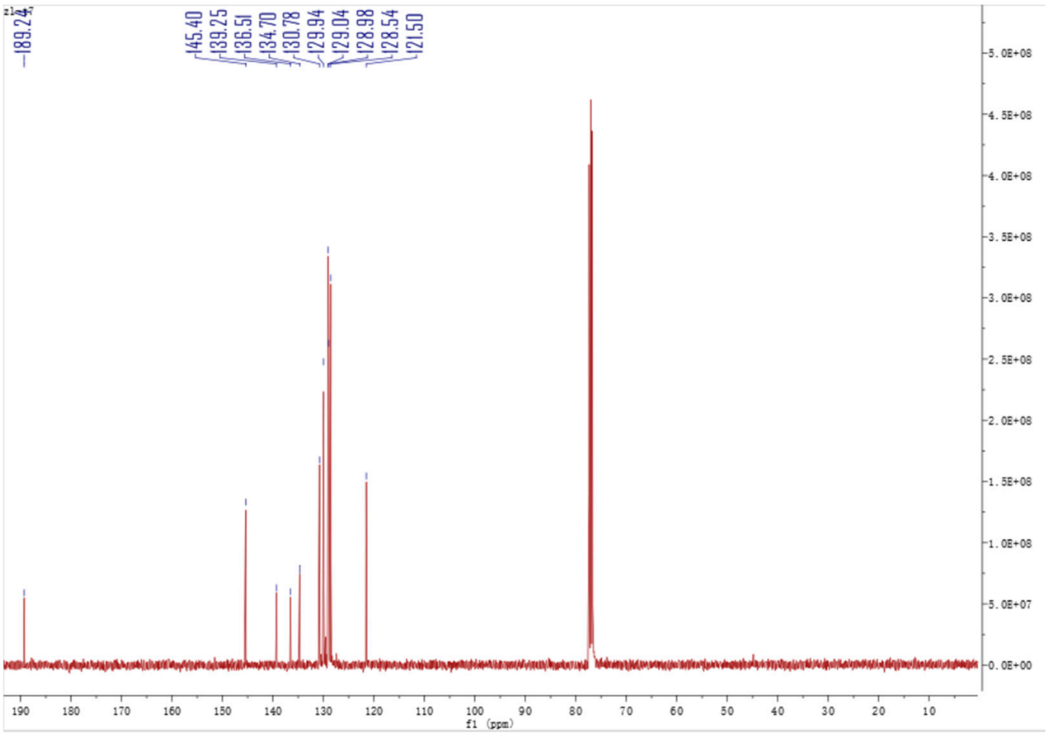

Scheme S13. ^{1}H-NMR and ^{13}C-NMR spectra of B'H_2 (R=Cl).

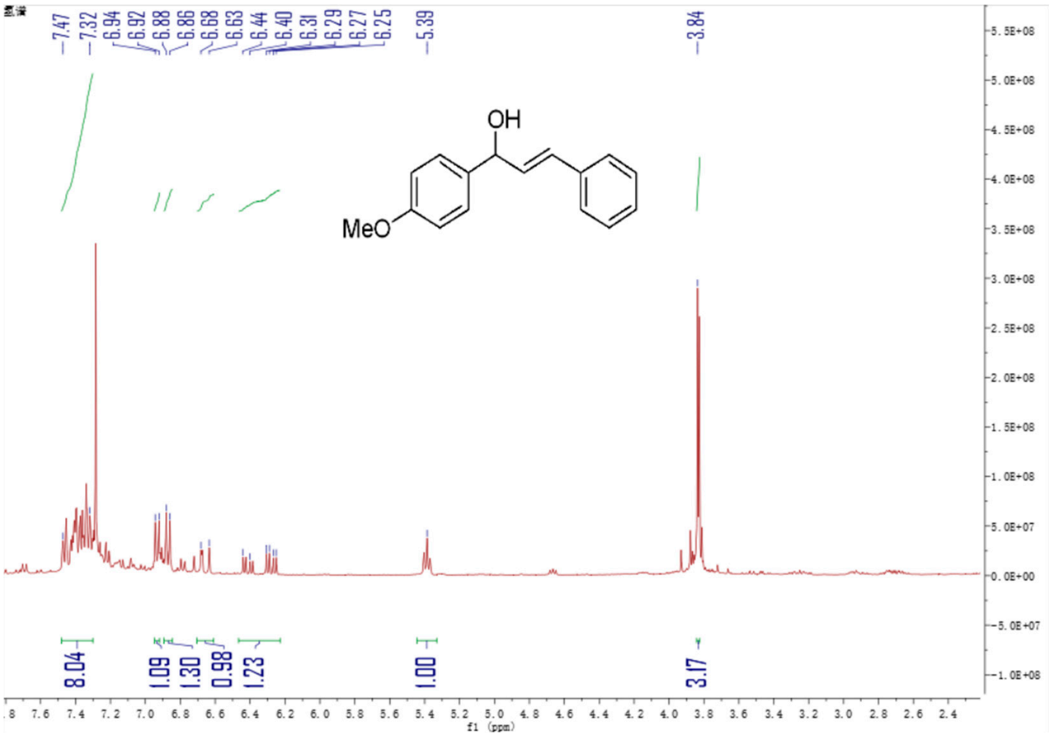

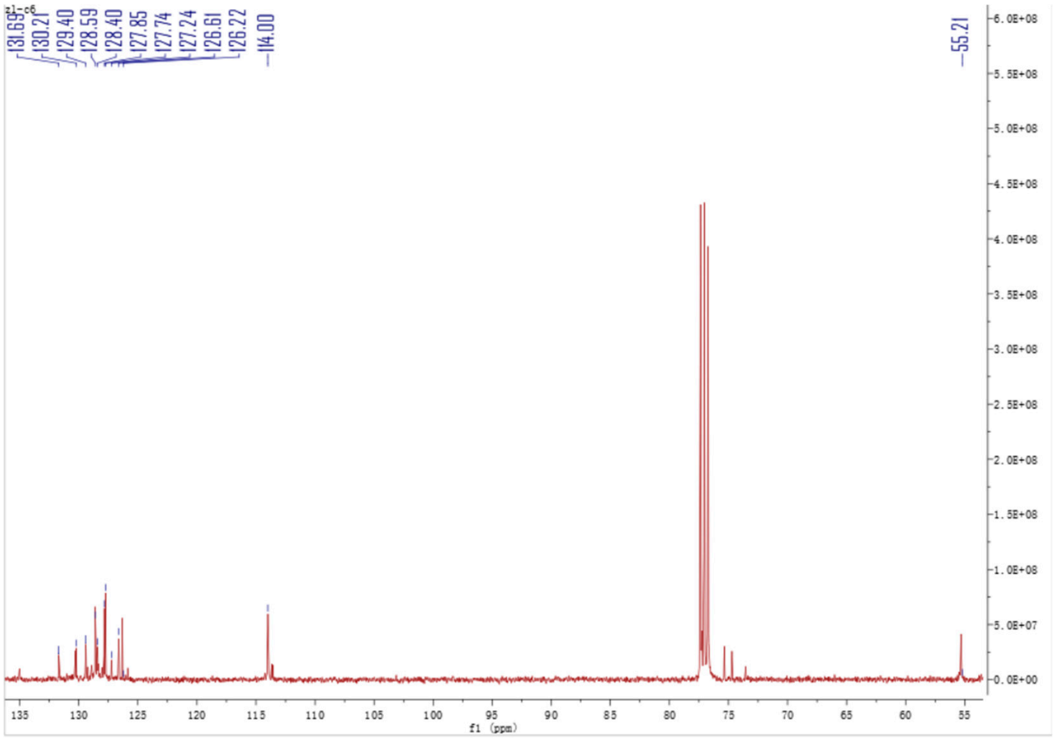

Scheme S14. ^{1}H-NMR and ^{13}C-NMR spectra of B'H_2 (R=OMe).

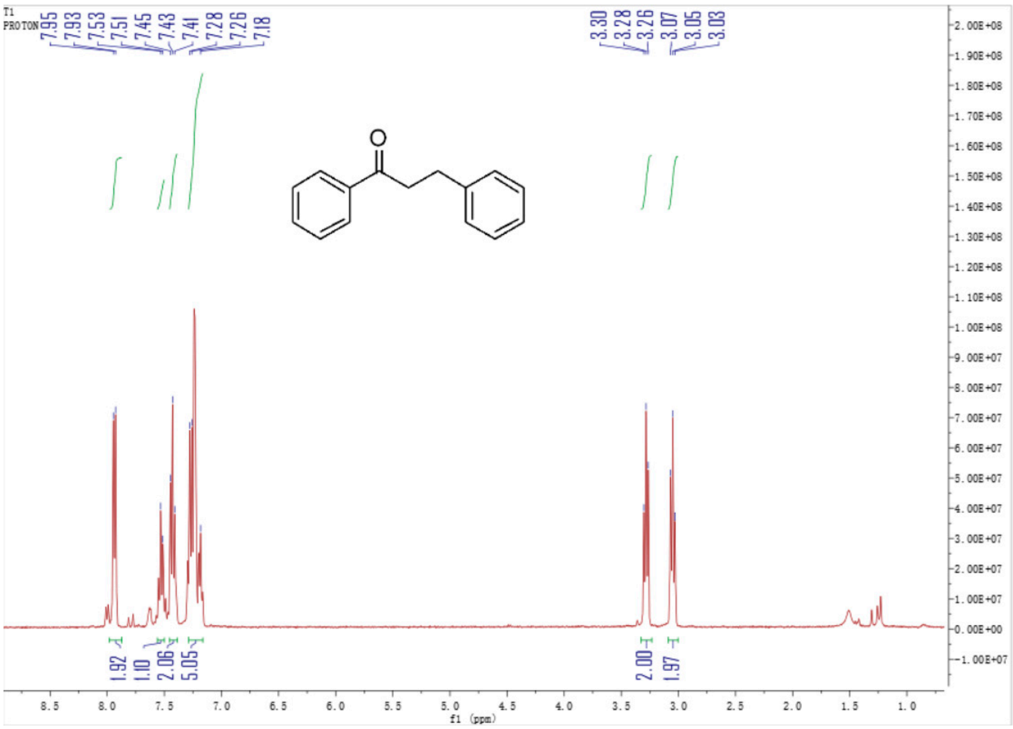

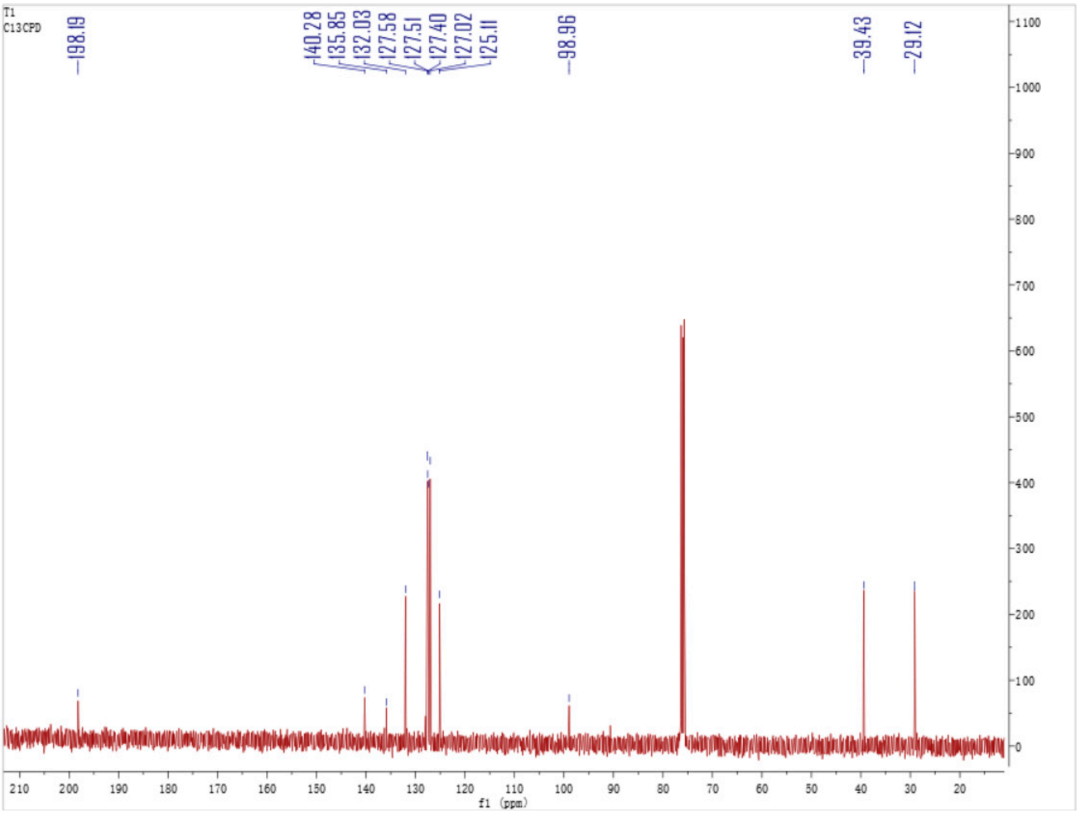

Scheme S15. ^{1}H-NMR and ^{13}C-NMR spectra of AH_2 (R=H).

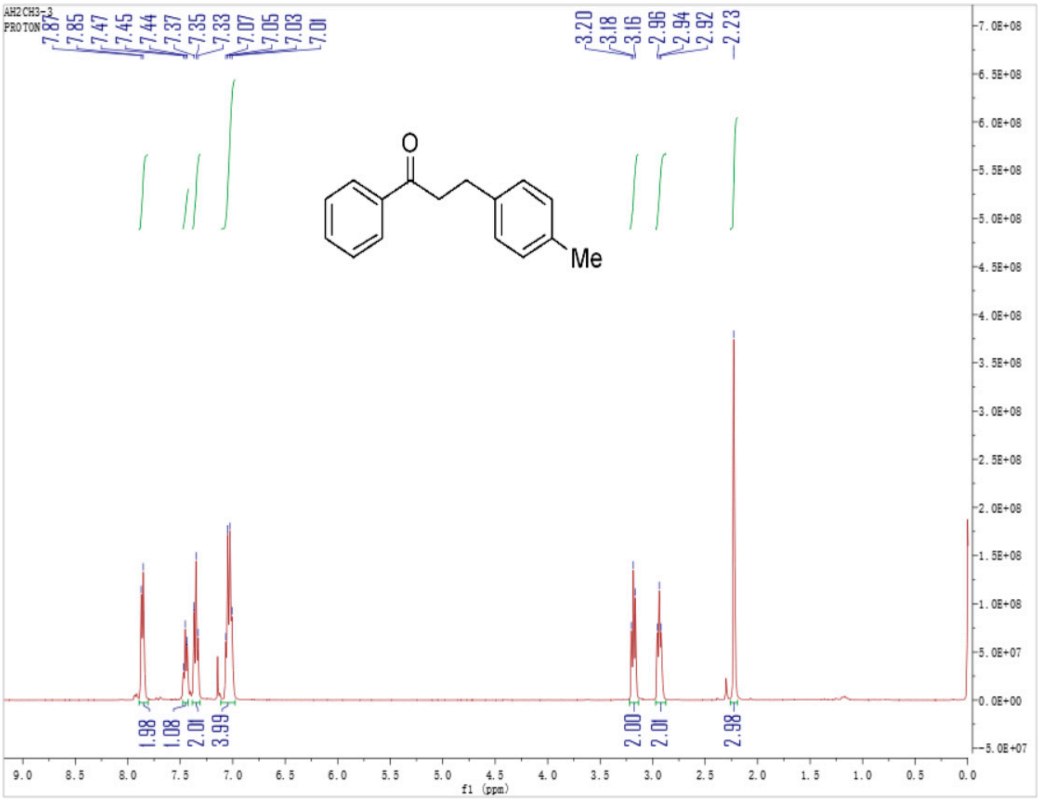

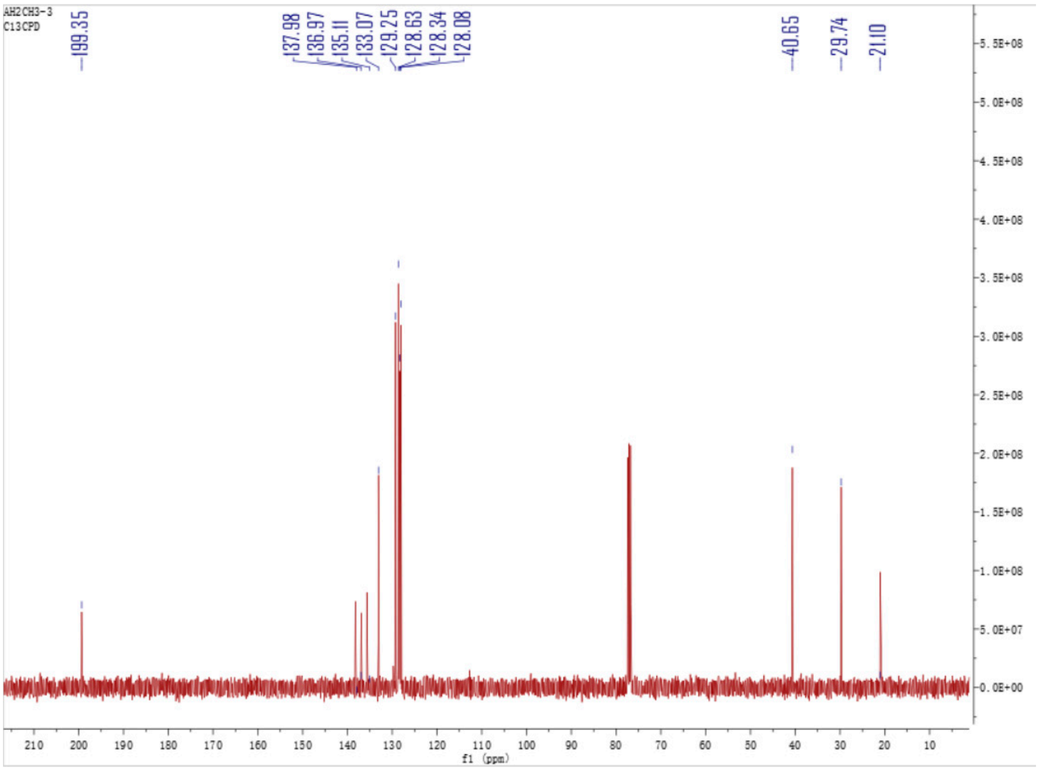

Scheme S16. ^{1}H-NMR and ^{13}C-NMR spectra of AH_2 (R=Me).

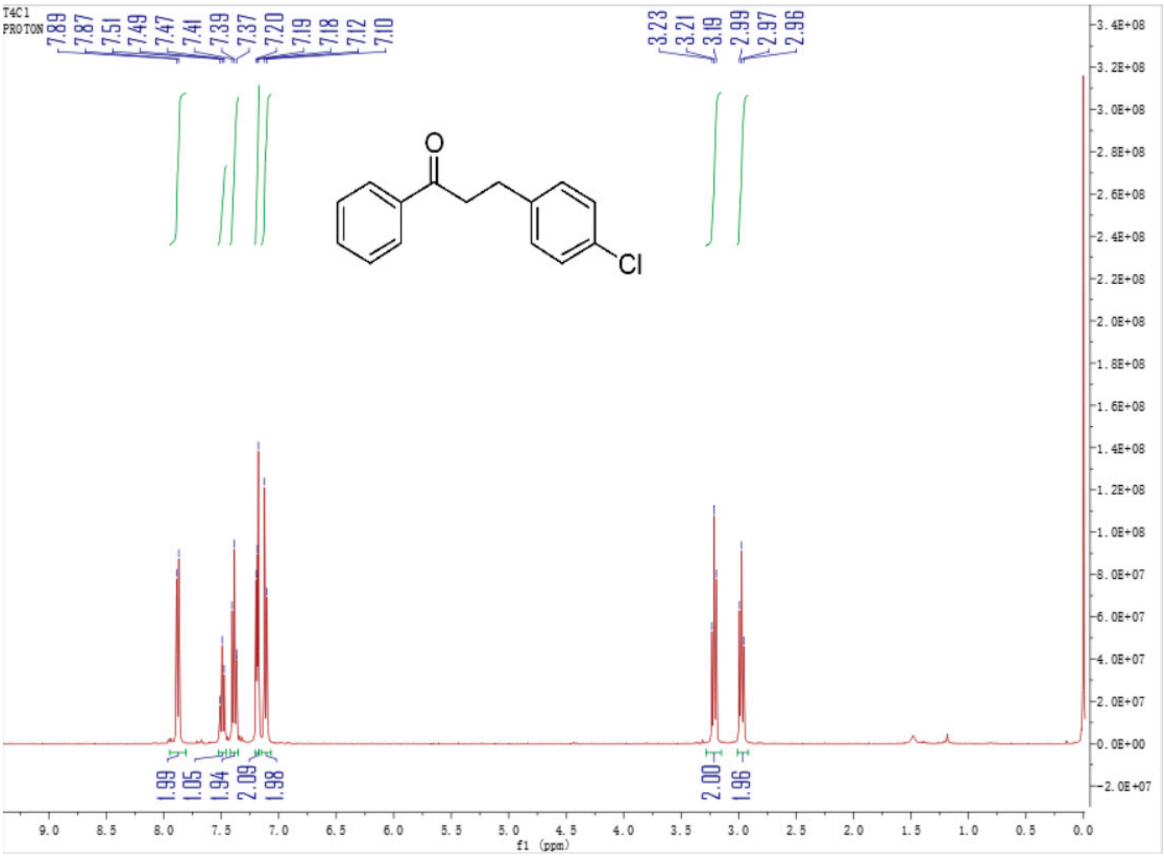

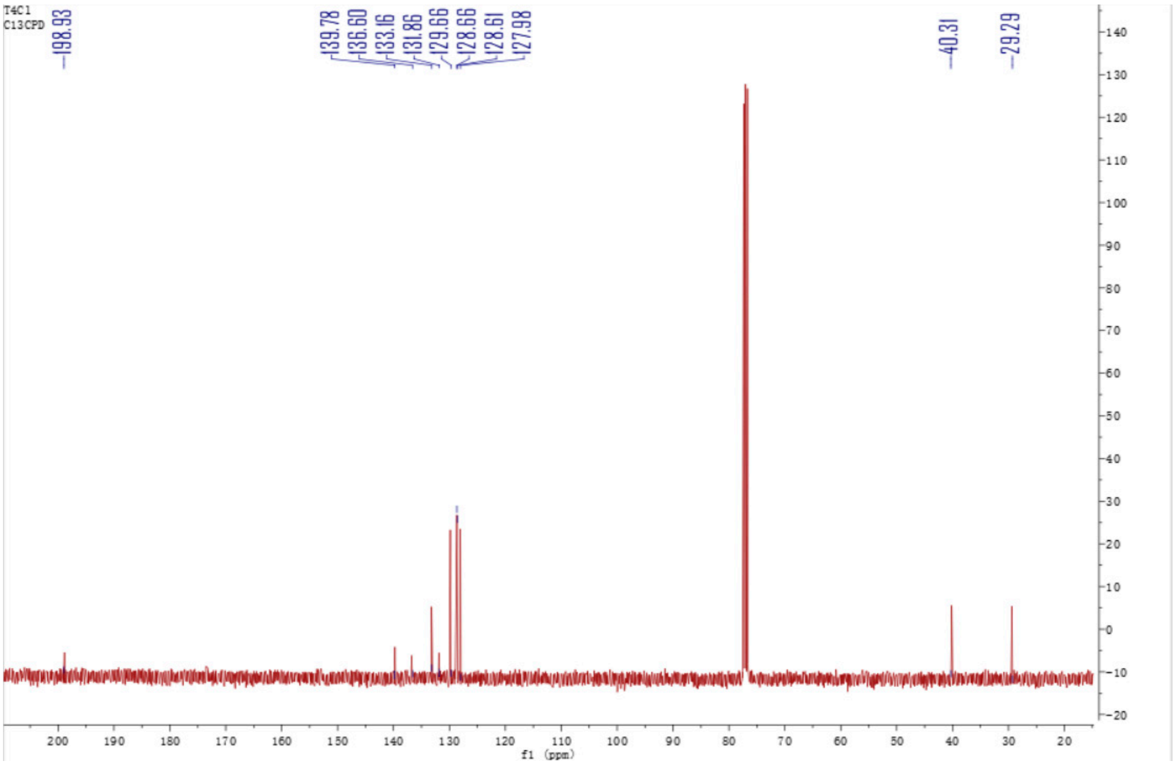

Scheme S17. ^{1}H-NMR and ^{13}C-NMR spectra of AH_2 (R=Cl).

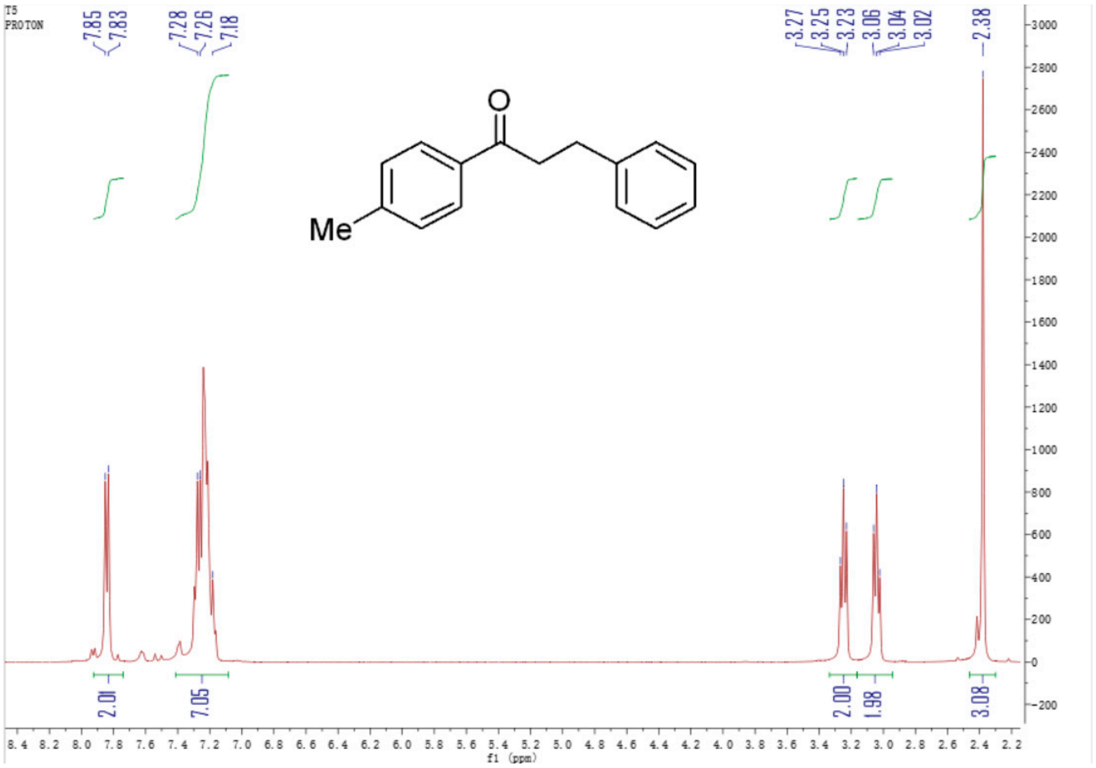

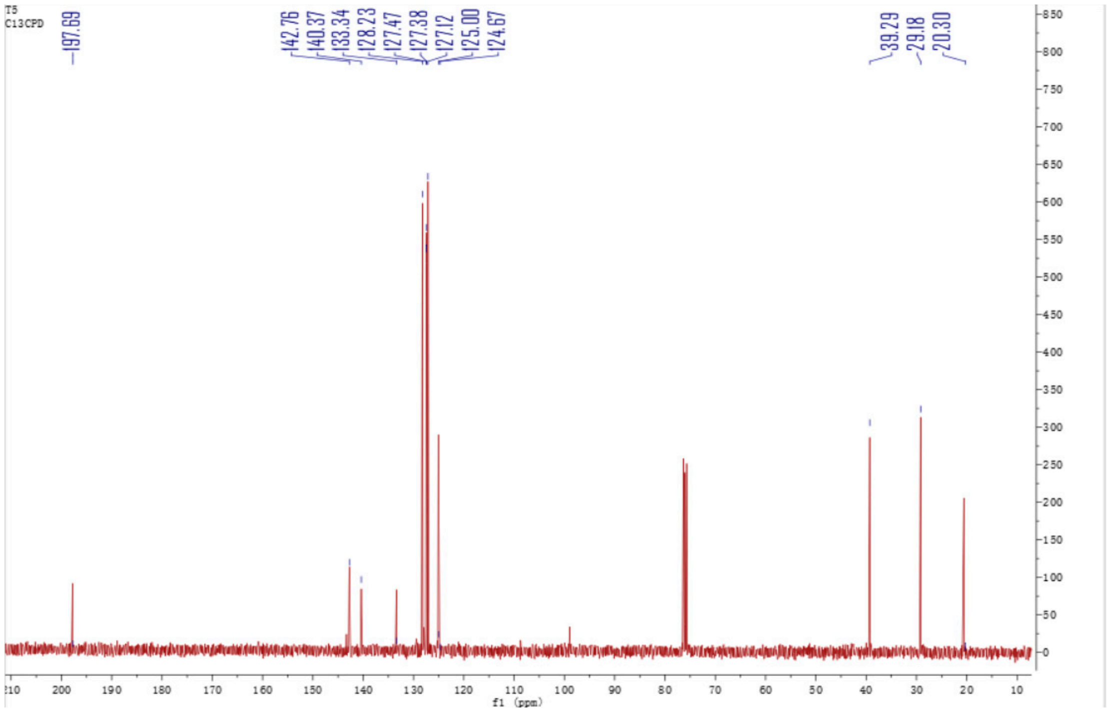

Scheme S18. ^{1}H-NMR and ^{13}C-NMR spectra of BH_2 (R=Me).

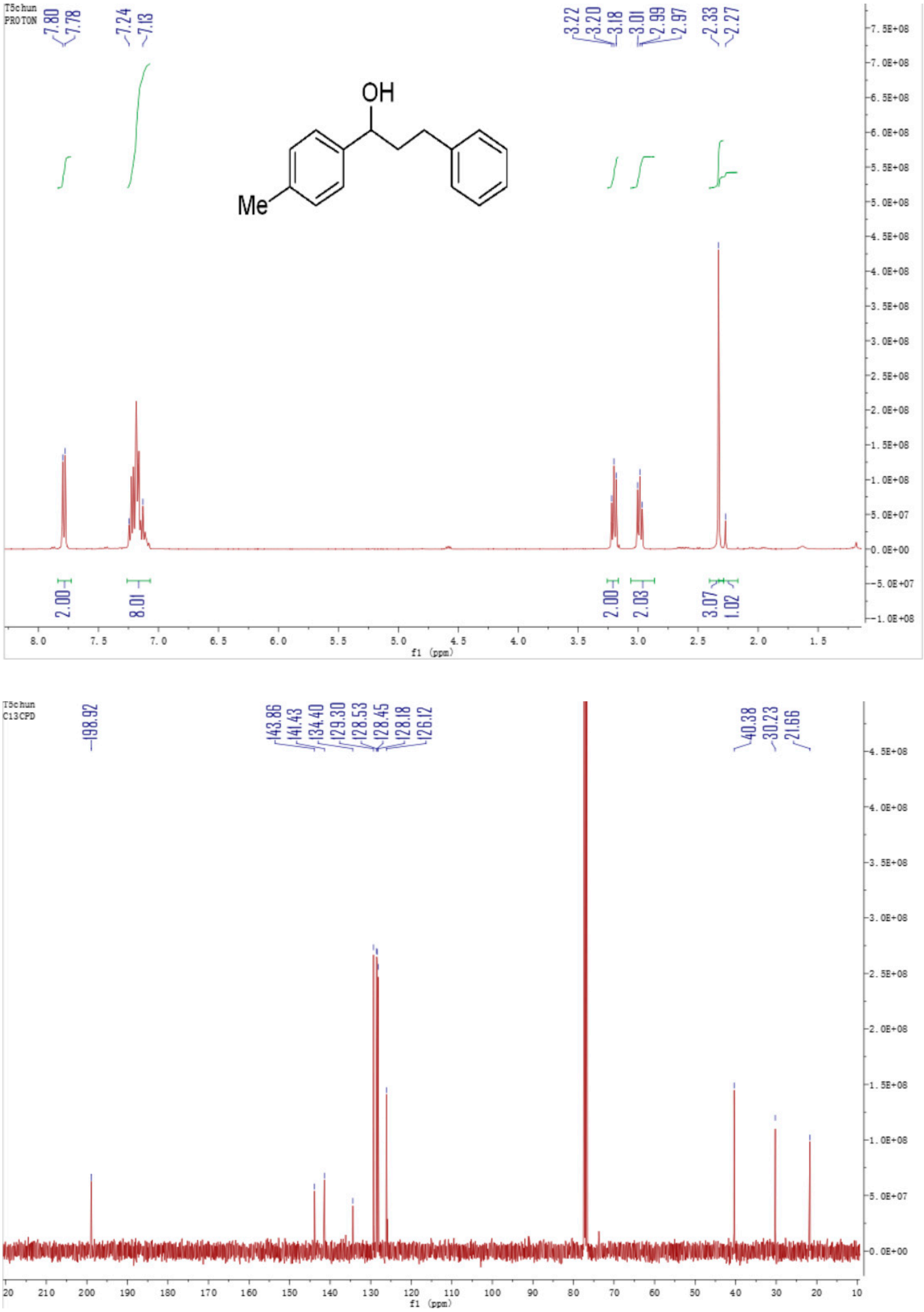

Scheme S19. ¹H-NMR and ¹³C-NMR spectra of BH_4 (R=Me).

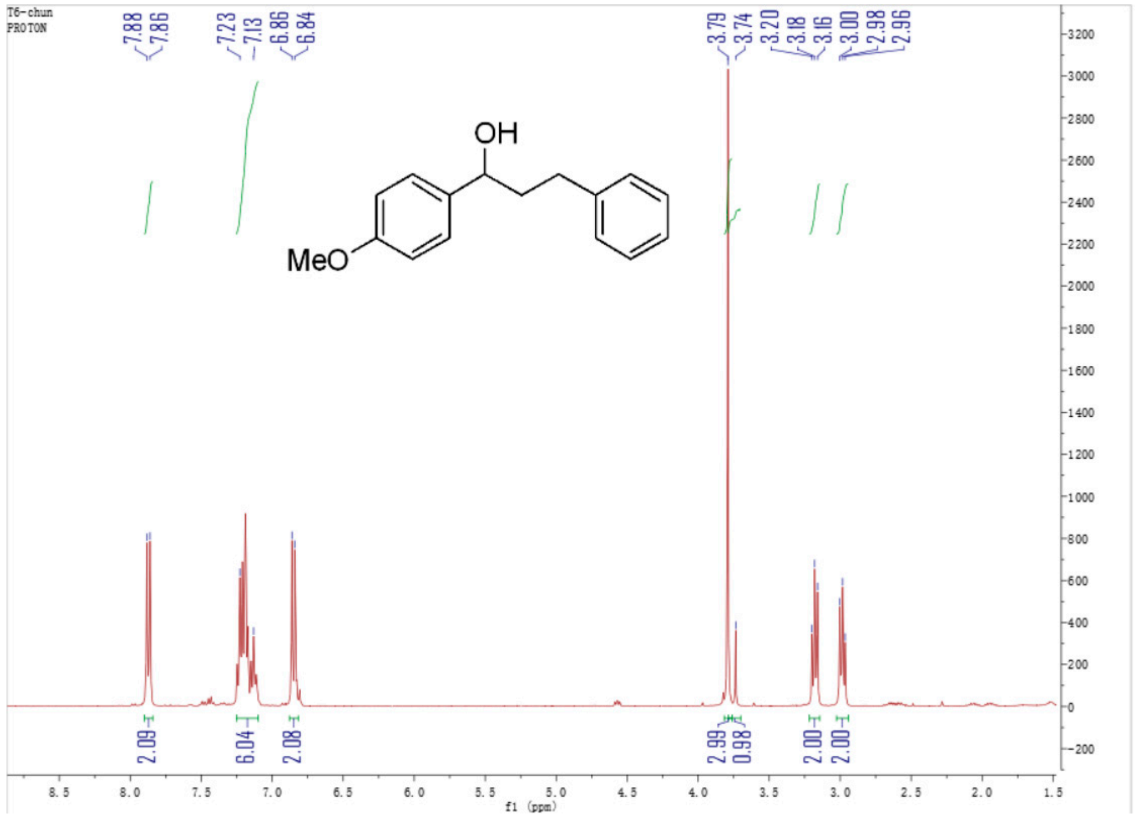

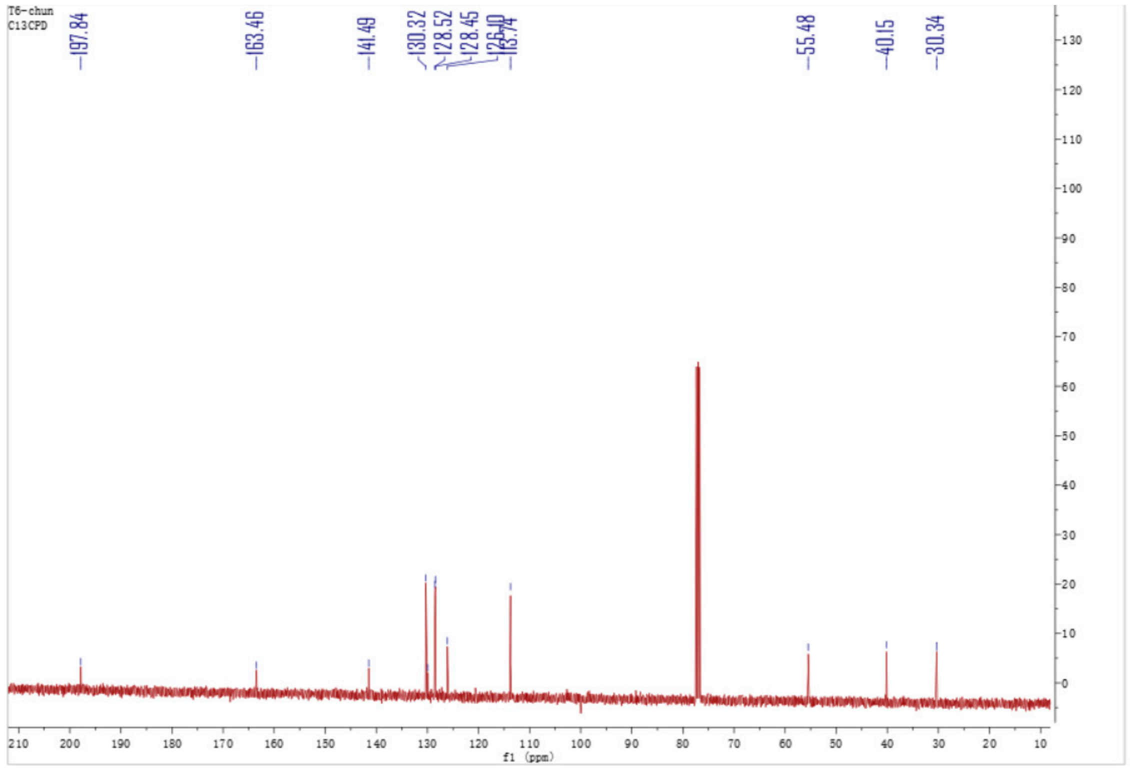

Scheme S20. ^{1}H-NMR and ^{13}C-NMR spectra of BH_4 (R=OMe).

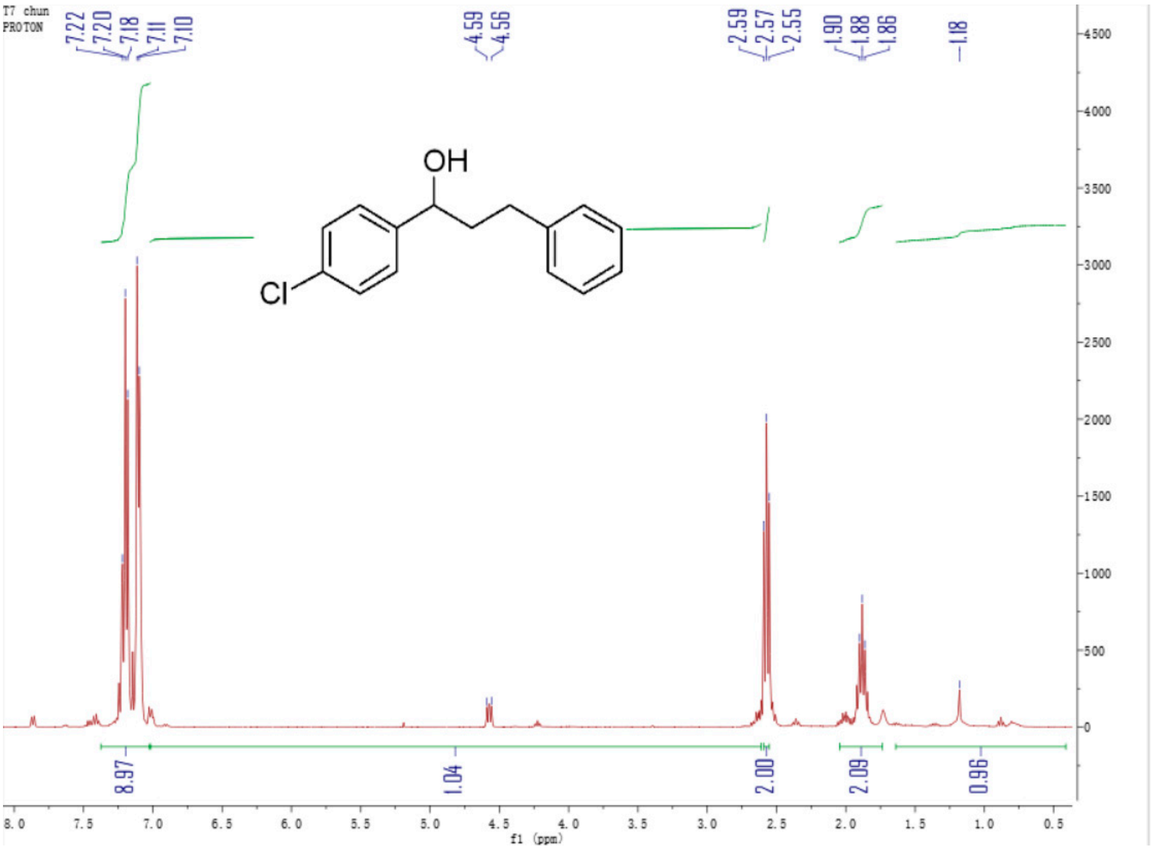

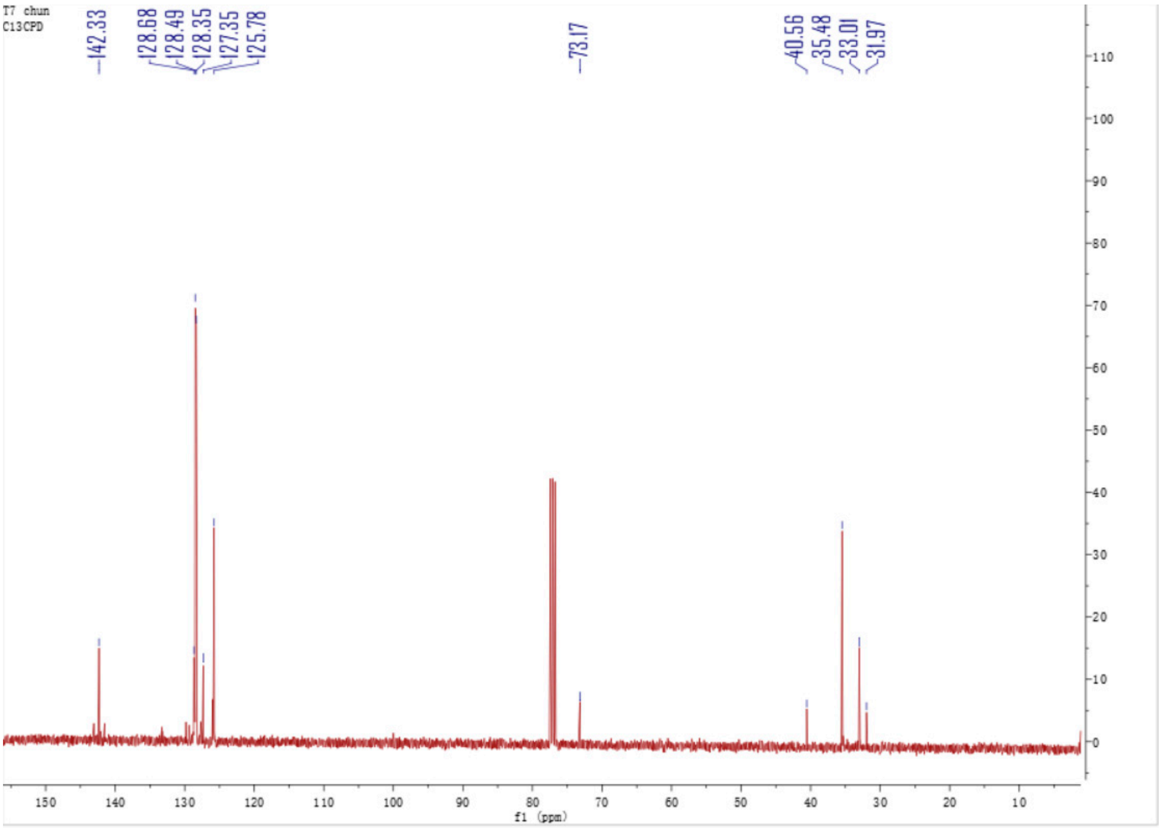

Scheme S21. ^{1}H-NMR and ^{13}C-NMR spectra of BH_4 (R=Cl).

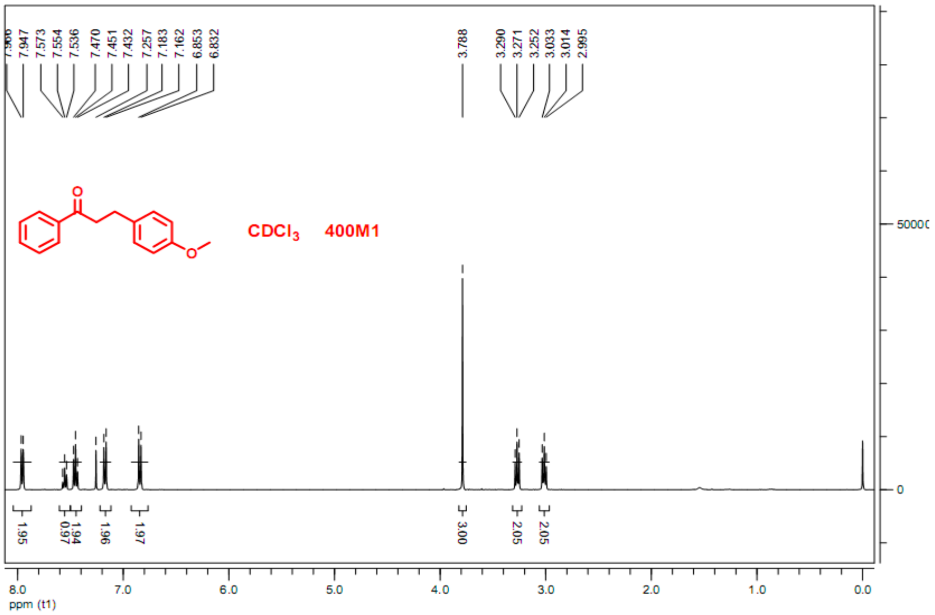

Scheme S22. ^{1}H-NMR spectra of AH_2 (R=OMe).

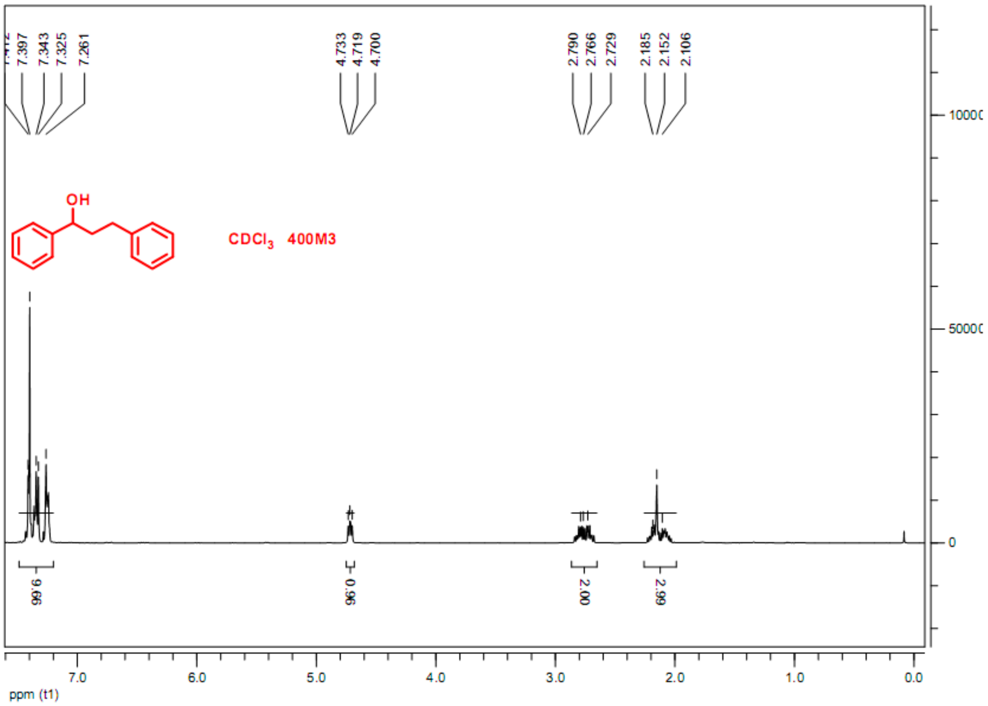

Scheme S23. ^{1}H-NMR spectra of AH_4 (R=H).

SV. Electrochemical spectra of representative compounds

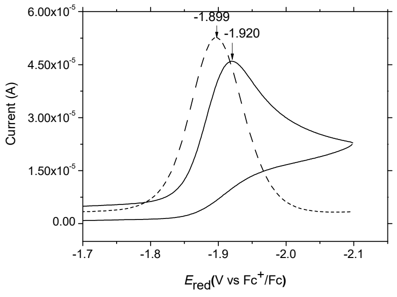

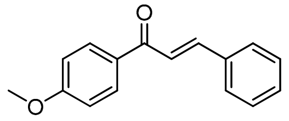

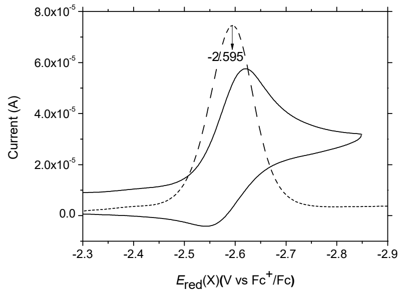

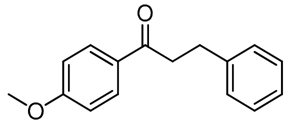

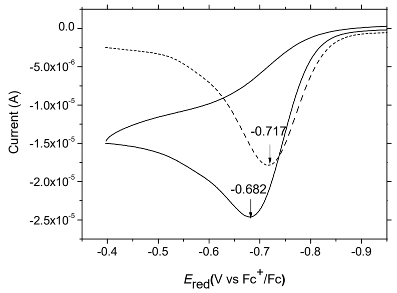

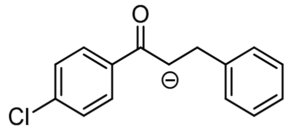

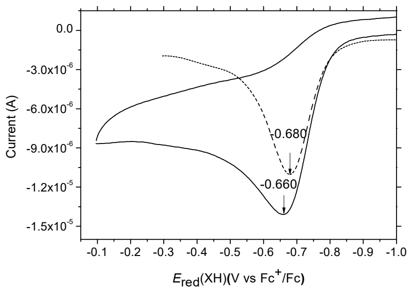

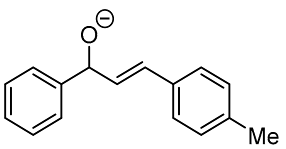

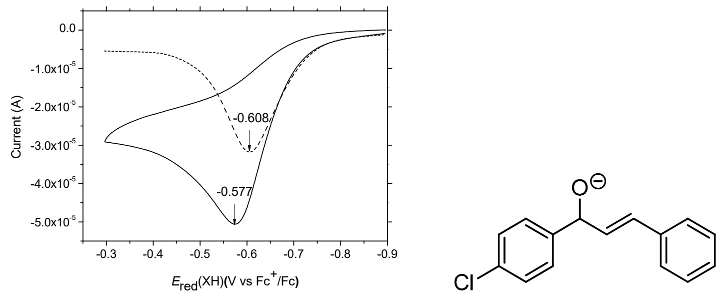
Current (A)
0.0
-1.0x10-5
-2.0x10-5
-3.0x10-5
-4.0x10-5
-5.0x10-5
-0.608
-0.577
-0.3
-0.4
-0.5
-0.6
-0.7
-0.8
-0.9
Ered(XH)(V vs Fc+/Fc)
Cl
O

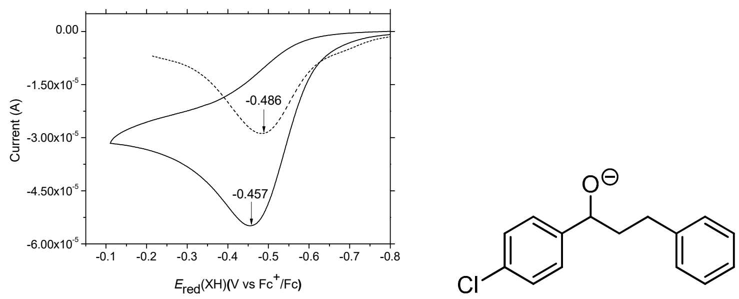
Current (A)
0.00
-1.50x10-5
-3.00x10-5
-4.50x10-5
-6.00x10-5
-0.486
-0.457
-0.1
-0.2
-0.3
-0.4
-0.5
-0.6
-0.7
-0.8
Ered(XH)(V vs Fc+/Fc)
Cl
O

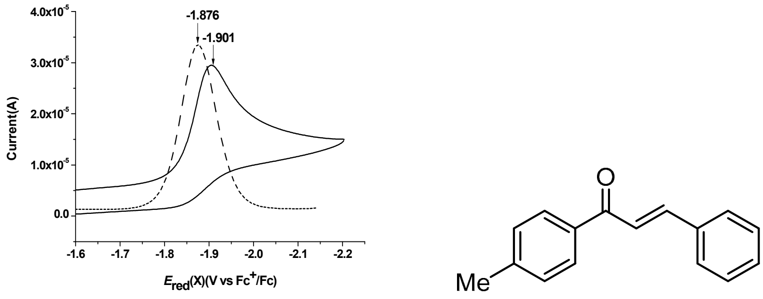
Current(A)
4.0x10-5
3.0x10-5
2.0x10-5
1.0x10-5
0.0
-1.876
-1.901
-1.6
-1.7
-1.8
-1.9
-2.0
-2.1
-2.2
Ered(X)(V vs Fc+/Fc)
Me
O

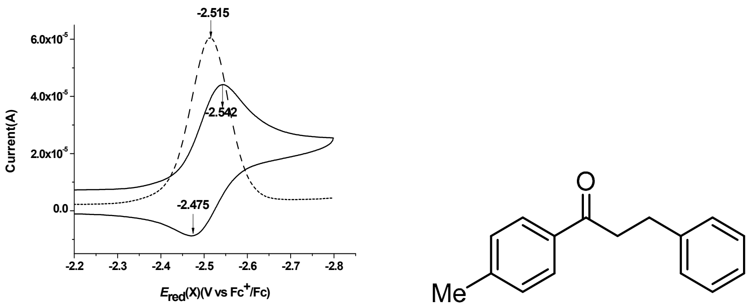
Current(A)
6.0x10-5
4.0x10-5
2.0x10-5
0.0
-2.515
-2.542
-2.475
-2.2
-2.3
-2.4
-2.5
-2.6
-2.7
-2.8
Ered(X)(V vs Fc+/Fc)
Me
O

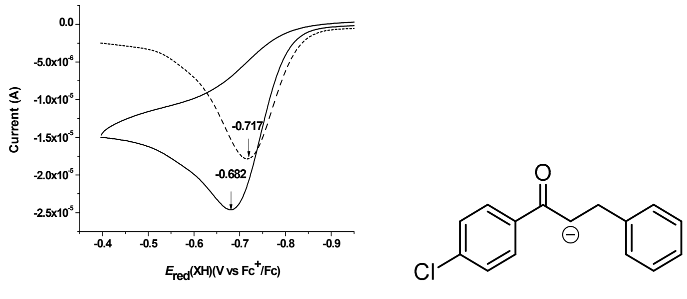
Current (A)
0.0
-5.0x10-6
-1.0x10-5
-1.5x10-5
-2.0x10-5
-2.5x10-5
-0.717
-0.682
-0.4
-0.5
-0.6
-0.7
-0.8
-0.9
Ered(XH)(V vs Fc+/Fc)
Cl
O

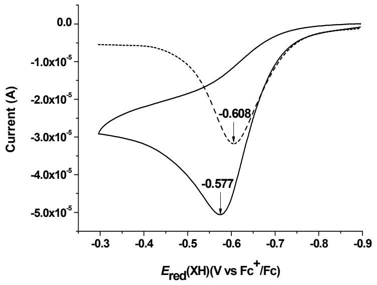

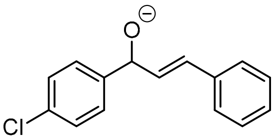

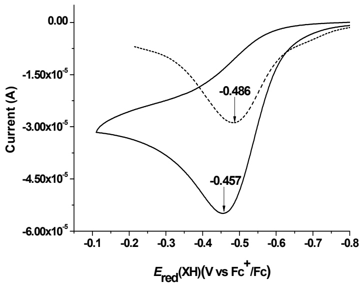

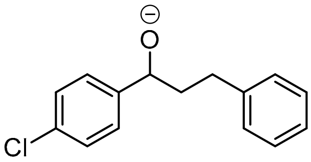

Scheme S24. Electrochemical spectra of 11 representative compounds.

SVI. ITC spectra of representative reactions

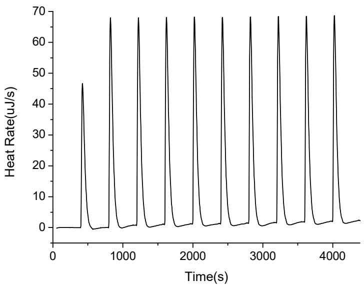

BNAH+TEMPO$^+$

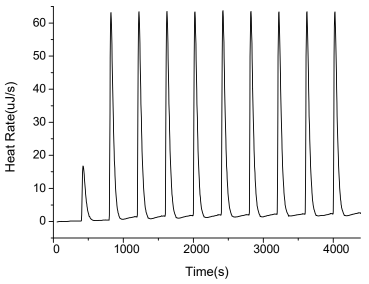

AH_2(R=H) (C$^-$)+TEMPO$^+$

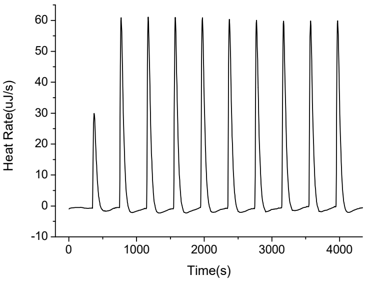

BH_2(R=OCH_3)(C$^-$)+TEMPO$^+$

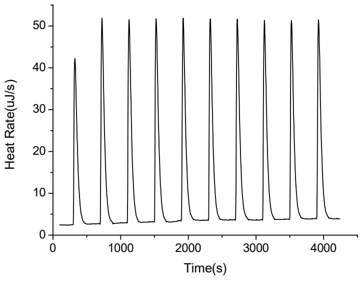

A’H_2(R=CH_3)(O$^-$)+TEMPO$^+$

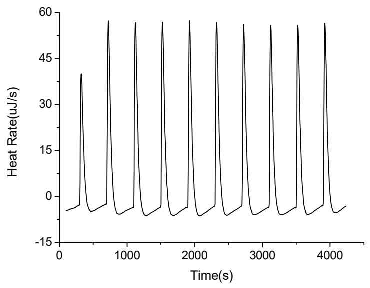

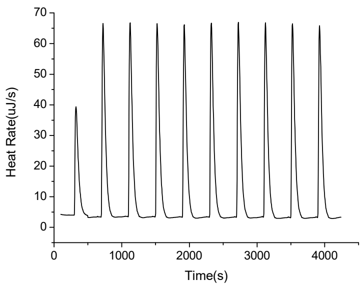

B'H_2(R=OCH_3)(O^-)+$TEMPO^+$ B'H_2(R=CH_3)(O^-)+$TEMPO^+$

Scheme S25. ITC spectra of 6 representative reactions
